# Genome-wide identification and expression analysis of the EXO70 gene family in grape (*Vitis vinifera* L)

**DOI:** 10.7717/peerj.11176

**Published:** 2021-04-21

**Authors:** Han Wang, Zong-Huan Ma, Juan Mao, Bai-Hong Chen

**Affiliations:** Department of Horticulture, Gansu Agricultural University, Lanzhou, China

**Keywords:** EXO70 gene, Grape, Gene expression, qRT-PCR

## Abstract

EXO70 is the pivotal protein subunit of exocyst, which has a very crucial role in enhancing the shielding effect of the cell wall, resisting abiotic and hormonal stresses. This experiment aims to identify family members of the *EXO70* gene family in grape and predict the characteristics of this gene family, so as to lay the foundation of further exploring the mechanism of resisting abiotic and hormone stresses of *VvEXO70s*. Therefore, the *Vitis vinifera* ‘Red Globe’ tube plantlet were used as materials. Bioinformatics was used to inquire VvEXO70 genes family members, gene structure, system evolution, *cis*-acting elements, subcellular and chromosomal localization, collinearity, selective pressure, codon bias and tissue expression. All of VvEXO70s had the conserved pfam03081 domain which maybe necessary for interacting with other proteins. Microarray analysis suggested that most genes expressed to varying degrees in tendrils, leaves, seeds, buds, roots and stems. Quantitative Real-Time PCR (qRT-PCR) showed that the expression levels of all genes with 5 mM salicylic acid (SA), 0.1 mM methy jasmonate (MeJA), 20% PEG6000 and 4 °C for 24 h were higher than for 12 h. With 20% PEG6000 treatment about 24 h, the relative expression of *VvEXO70-02* was significantly up-regulated and 361 times higher than CK. All genes’ relative expression was higher at 12 h than that at 24 h after treatment with 7 mM hydrogen peroxide (H_2_O_2_) and 0.1 mM ethylene (ETH). In conclusion, the expression levels of 14 *VvEXO70* genes are distinguishing under these treatments, which play an important role in the regulation of anti-stress signals in grape. All of these test results provide a reference for the future research on the potential function analysis and plant breeding of *VvEXO70* genes.

## Introduction

The yield and quality of plants under biotic and abiotic stresses are seriously damaged, so it is essential to explore the defense mechanism (Bu et al., 2019). When plants are under biotic and abiotic stresses, the signaling pathway related to receptor proteins is activated and exocytosis occurs, which enhances the shielding effect of cell wall (Yun & Kwon, 2017). Exocytosis refers to the process of the transport vesicles derived from the post-golgi transport membrane fuse with the target membrane pass through certain transport pathways (Jurgens & Geldner, 2002). It plays a very important role in all eukaryotic cells, including cell growth, cell polarization, cell division, cell information transfer, the formation of the cell walls of the physiological process (Yao et al., 2013; Yang et al., 2013; Yang et al., 2015a; Yang et al., 2015b), especially has a crucial role in plant resistance mechanisms (Gu, Zavaliev & Dong, 2017).

EXO70 is one of the eight protein subunits in the exocrine complex, with the seven other ones (SEC3, SEC5, SEC6, SEC8, SEC10, SEC15 and EXO84), is involved in the tethering process of the membrane vesicles at the budding site. The tethering process is the third step (budding, transport, tethering, fusion) in the material exchange process of different membrane structures in the cell, which is involved in the initial contact process of the transport vesicles and the target membrane and is a key step in regulating extracellular secretion (TerBush & Novick, 1995; TerBush et al., 1996; Finger, Hughes & Novick, 1998; Guo, Grant & Novick, 1999; Whyte & Munro, 2002; Elias et al., 2003; Cai, Reinisch & Ferro-Novick, 2007). The C-terminal of EXO70 protein carries several negative charge residues (Zhu, Wu & Guo, 2019), and EXO70 and SEC3 can bind to phosphatidylinositol-4, 5-diphosphate (PIP2), which is involved in the anchoring of exocrine complex secretory tomont and target plasma membrane (Liu et al., 2018). Meanwhile, EXO70 can directly interact with Rho GTPase family members of small G protein, participating in the assembly and activation of SNARE protein and regulating the assembly of exocyst complex (Sivaram et al., 2005; He et al., 2007; Moore, Robinson & Xu, 2007; Wu et al., 2010; Chen et al., 2012; Ren & Guo, 2012).

The mechanism of exocyst was first identified in yeast studies. *EXO70* was localized at the activation site of vesicle-plasma membrane fusion in yeast (Mathieson et al., 2010). In animals, eight proteins of yeast-homologous exocrine complex were first isolated in the brain tissues of mice (Ma et al., 2016). *EXO70* gene families of yeast and animals have only one member, meanwhile, there are multiple members in *EXO70* gene family of plants, which shows the multi-copy phenomenon peculiar to plants (Table S1) (Moore, Robinson & Xu, 2007; Yang et al., 2013). For example, the genes encoding *SEC6*, *SEC8* and *SEC10* in the *Arabidopsis thaliana* genome have only one copy respectively, while the genes encoding *EXO70* have 23 copies. The genes encoding *SEC5*, *SEC6*, *SEC8* and *SEC10* in the rice genome had only one copy each, while the genes encoding *EXO70* had 47. This indicates that the replication of *EXO70* gene is unique to terrestrial higher plants (Li et al., 2010). The cause of this phenomenon has not been determined, and it is speculated that the *EXO70* genes in different organisms are involved in various biological processes (Bu et al., 2019). Studies have found that exocytosis of higher plants acts on plant growth and development (Jurgens & Geldner, 2002). Among the 23 *A. thaliana EXO70* genes, *AtEXO70A1* was involved in the growth of pollen tube and root hairs, resulting in shorter root and stigma hairs (Synek et al., 2006; Samuel et al., 2009). *AtEXO70C1* (*At5g13150*) gene mutated, resulting in delaying pollen tube development and blocking male transmission (Li et al., 2010). Moreover, *EXO70* is involved in pollen-stigma interactions in *Brassica* and the over-expression of *BrEXO70A1* is sufficient to overcome the self-pollination rejection of the species with partial self-incompatibility (Samuel et al., 2009; Zhang et al., 2010). During the development and maturation of *Glycine max* L, the expression of some *GmExo70J* genes including *GmExo70J1*, *GmExo70J6* and *GmExo70J7* increases greatly in floral organ-supporting receptacles, indicating a possible role in seed development (Wang et al., 2016).

At present, the *EXO70* gene in yeast and mammals has been widely studied, but only a few kinds of plants have been done, such as tomato (*Solanum lycopersicum*) had 22, potato (*Solanum tuberosum*) had 21, tobacco (*Nicotiana benthamiana*) had 44 (Yu et al., 2018), *A. thaliana* had 23 (Li et al., 2010), Chinese cabbage (*Brassica pekinensis*) had 39, cabbage (*Brassica oleracea*) had 45 (Yang et al., 2015b), diploid tobacco had 24 (Juraj et al., 2017), wheat (*Triticum aestivum*) had 200 (Zhao et al., 2018) *EXO70* genes. However, the study of EXO70 gene families in grape is rarely reported. Besides, genome-wide identification and expression analysis are effective ways to clarify the classification and composition of gene family members in genome, which is the primary task to explore biological issues related to species characteristics. These can lay a foundation for subsequent functional studies and genetic manipulation of gene. Therefore, in this study, a genome-wide identification and expression analysis of *VvEXO70* gene family was conducted and bioinformatics was used to inquire *VvEXO70* gene family members, genetic structure, system evolution and cis-acting element, subcellular and chromosomal localization, collinearity, selective pressure, codon bias and tissue expression. By establishing the simulation environment of different treatment for grape test-tube seedlings and analyzing the relative expression quantity, it could provide the reference for the further research of the function of the gene family and the breeding of resistant varieties in grape.

## Materials and Methods Introduction

### Overview of study

**Figure 1:**
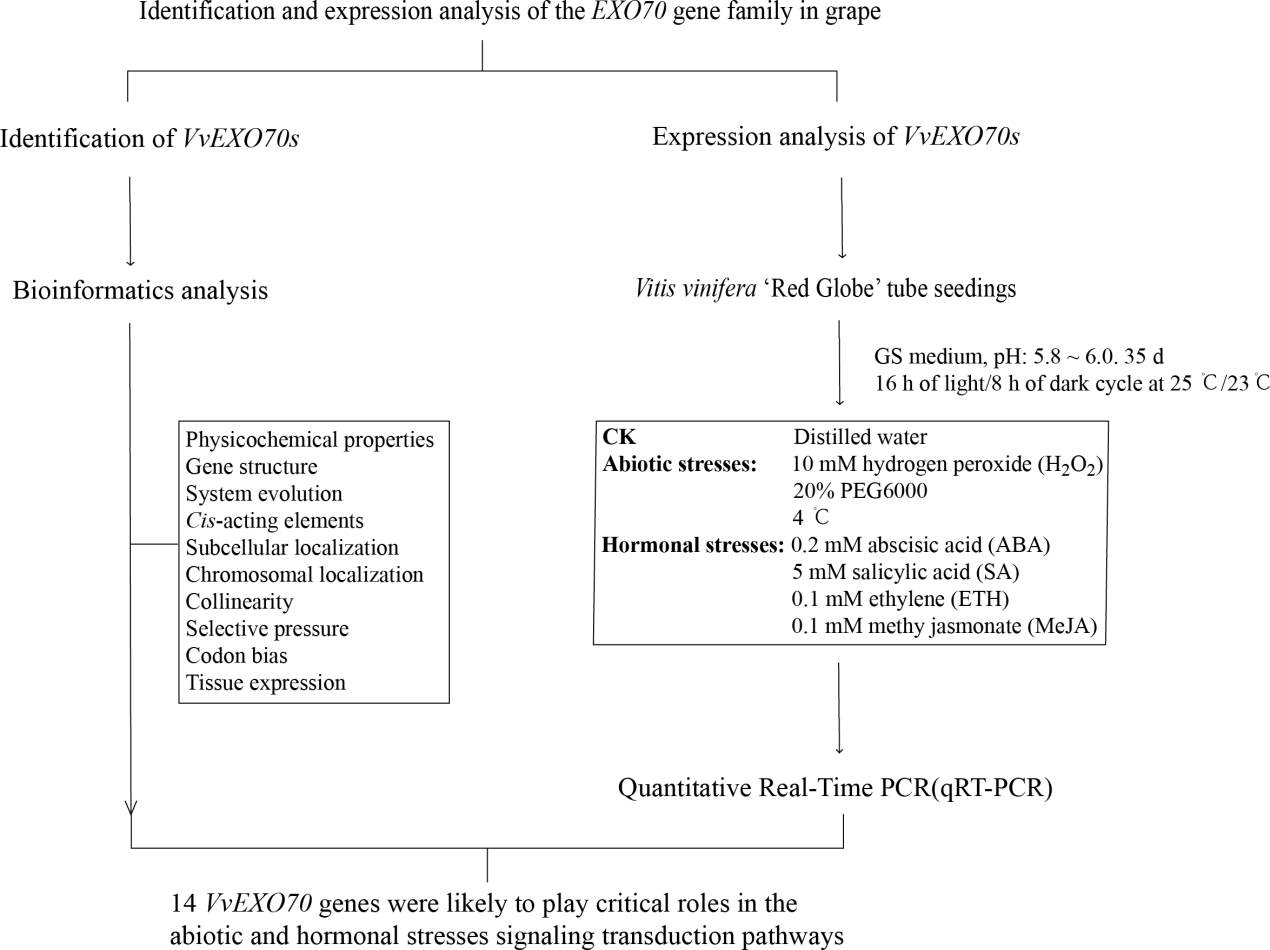
The research route of this study. The experiment mainly included genome-wide identification and expression analysis of *EXO70* gene family in grape to preliminarily predict the function of *VvEXO70s*.

As shown in Fig. 1, the experiment mainly included genome-wide identification and expression analysis of *EXO70* gene family in grape to preliminarily predict the function of *VvEXO70s*. On the one hand, we identified the members of *VvEXO70s* by bioinformatics and on the other hand, we analyzed the responses of this gene family to some abiotic stresses by qRT-PCR. These test results could provide a reference for the future research on the potential function analysis and plant breeding of *VvEXO70* genes.

### Plant materials and treatments

The experiment materials were *Vitis vinifera* ‘Red Globe’ tube seedings, which were stored in College of Horticulture, Gansu Agricultural University. At the beginning of the test, we placed stem-segment with single bud of tube seedlings on ordinary GS medium and pH was 5.8∼6.0. Materials were cultured in the incubator under a 16 h of light/8 h of dark cycle at 25 °C/23 °C. Different stress treatments were conducted about 35 d seedling plants. For abiotic stress treatments, the seedlings were incubated in a solution containing 20% PEG6000, 10 mM hydrogen peroxide (H_2_O_2_) and the low temperature treatment condition is 4 °C. For phytohormone treatments, the seedlings were cultured in GS medium with 5 mM salicylic acid (SA), 0.1 mM methy jasmonate (MeJA), 0.1 mM ethylene (ETH) and 0.2 mM abscisic acid (ABA) respectively, meanwhile, the volume of distilled water as a CK. Every stress repeated three times. Then we collected leaves from each treatment about 12 h and 24 h. All of the collected materials were stored at −80 °C for RNA extraction and gene expression analysis.

### Identification of EXO70 genes family members in grape and analysis of physicochemical properties

*A. thaliana* IDs (Li et al., 2010; Zhao et al., 2018) were used to download amino acid sequences of each gene from TAIR database (https://www.arabidopsis.org). After that amino acid sequences were used to homologously search *EXO70* genes family in grape genome website (http://www.genoscope.cns.fr/) and rice genome website (http://rice.plantbiology.msu.edu/). We could screen genes by judging whether they include the functional domain ‘PF03081.15’ on HMMER (https://www.ebi.ac.uk/Tools/hmmer/). Meanwhile, DNAMAN software could do multiple sequence alignment. ExPASy (https://web.expasy.org/protparam/) was used to acquire some basic physicochemical properties about *VvEXO70s* such as the number of exon, molecular weight (MW), isoelectric point (pI), instability index (I.I), aliphatic index (A.I), grand average of hydropathicity (GRAVY).

### Bioinformatics analysis of the VvEXO70s

Clustal X, MEGA7.0 and EvolView (https://www.evolgenius.info/) were used to constructed a phylogenetic tree of grape, rice and *A. thaliana EXO70* genes by the basis of the neighbor-joining method. The bootstrap analysis was performed using 1,000 repetitions. The gene structure of the *VvEXO70s* was detected by using GSDS 2.0 (http://gsds.cbi.pku.edu.cn/) based on their corresponding amino acid sequences. The secondary structure was identified on NPS@:SOPMA (https://npsa-prabi.ibcp.fr/cgi-bin/npsa_automat.pl?page=npsa_sopma.html). The 3D structure was created with SWISS-MODEL (https://swissmodel.expasy.org/). The chromosomal location information of *VvEXO70s* was obtained from the grape genome website (http://www.genoscope.cns.fr/) and was drawn a picture by MG2C (http://mg2c.iask.in/mg2c_v2.0/). MEME (http://meme-suite.org/tools/meme) was used to analyze the conserved motifs of VvEXO70 proteins. Subcellular localization analysis was conducted on WoLF PSORT (https://www.genscript.com/wolf-psort.html). PLACE (https://www.dna.affrc.go.jp/PLACE/?action=newplace) was used to analyze the distribution of *cis*-acting elements of the 2 kb of upstream of 14 *VvEXO70* genes.

### Collinearity and selective pressure analysis VvEXO70s

Online software (http://tools.bat.infspire.org/circoletto/) was used to analyze the collinearity of *EXO70* genes between grape and *A. thaliana*.

Pairs of genes with collinearity would be done sequence alignment by using Clustal Omega (https://www.ebi.ac.uk/Tools/msa/clustalo/). PAL2NAL (http://www.bork.embl.de/pal2nal/index.cgi?) was used to calculate the non-synonymous/synonymous (d_N_/d_S_) value of duplicate gene pairs (Goldman & Yang, 1994).

### Codon usage bias analysis VvEXO70s

Codon composition and usage preference of *VvEXO70* gene family members were analyzed by CodonW software. Relative synonymous codon usage (RSCU) refers to the ratio of the frequency used by a particular codon to the frequency expected in unbiased use. Measures of codon use preference include: RSCU, number of valid codons (ENC), codon adaptation index (CAI), and codon bias index (CBI). Measures indicators of codon composition include: the occurrence frequency of adenine, thymine, guanine, and cytosine at position 3 (A3s, T3s, G3s, C3s), frequency of optimal codons (FOP), Guanine and cytosine content (GC content), GC content at the third site of the synonymous codon (GC3s content), aromatic amino acid frequency (Aromo), synonymous codon number (L_sym), the total number of synonymous and non-synonymous codons (L_aa), protein hydrophilicity (Gravy). Correlation analysis between codon composition and preference parameters was carried out by using SPSS 23.0 statistical software.

### Tissue-specific expression patterns of VvEXO70s

BLAST program on Ensembl Plants (http://plants.ensembl.org/index.html) was used to find the accession numbers of tissue-specific expression of *VvEXO70s*. These ids were used to gain the tissue-specific expression data related to the different tissues at various developmental stages of grape on BAR (https://bar.utoronto.ca/). R language was used to visualize the results.

### RNA Isolation and qRT-PCR analysis of VvEXO70s

The RNA were extracted from plant leaves by using a Spectrum Plant Total RNA kit (Sigma St. Louis, MO, USA) following the operating instructions.

The primer design and synthetic were accomplished in Sangon Biotech in Shanghai. The Reverse Transcriptase M-MLV (RNase H-) kit (TaKaRa Biotechnology. Lanzhou, China) was used to synthesize cDNA. Light Cycler^®^ 96 Real-Time PCR System (Roche, Basel, Switzerland) was used to perform qRT-PCR of VvEXO70s. The gene primers (Table S2) were designed in Sangon and used for PCR amplification, among which GAPDH gene (GenBank accession no. CB973647) was internal control genes. The amplification volume was 25 µL containing 1 µL forward primer, 1 µL reverse primer, 1.5 µL cDNA, 9 µL ddH_2_O and 12.5 µL TaKaRa SYBR Premix Ex Taq. II (TaKaRa Biotechnology. Lanzhou, China). The response procedures was: 95 °C for 30 s, 40 cycles of 95 °C for 5 s, and 60 °C for 30 s. For melting curve analysis, a program including 95 °C for 15 s, followed by a constant increase from 60 °C to 95 °C, was included following the PCR cycles. Each treatment was run three biological replicates. The 2^−ΔΔ^CT^^ method was used to calculate the relative expression levels of genes (Udvardi, Czechowski & Scheible, 2008).

**Figure 2:**
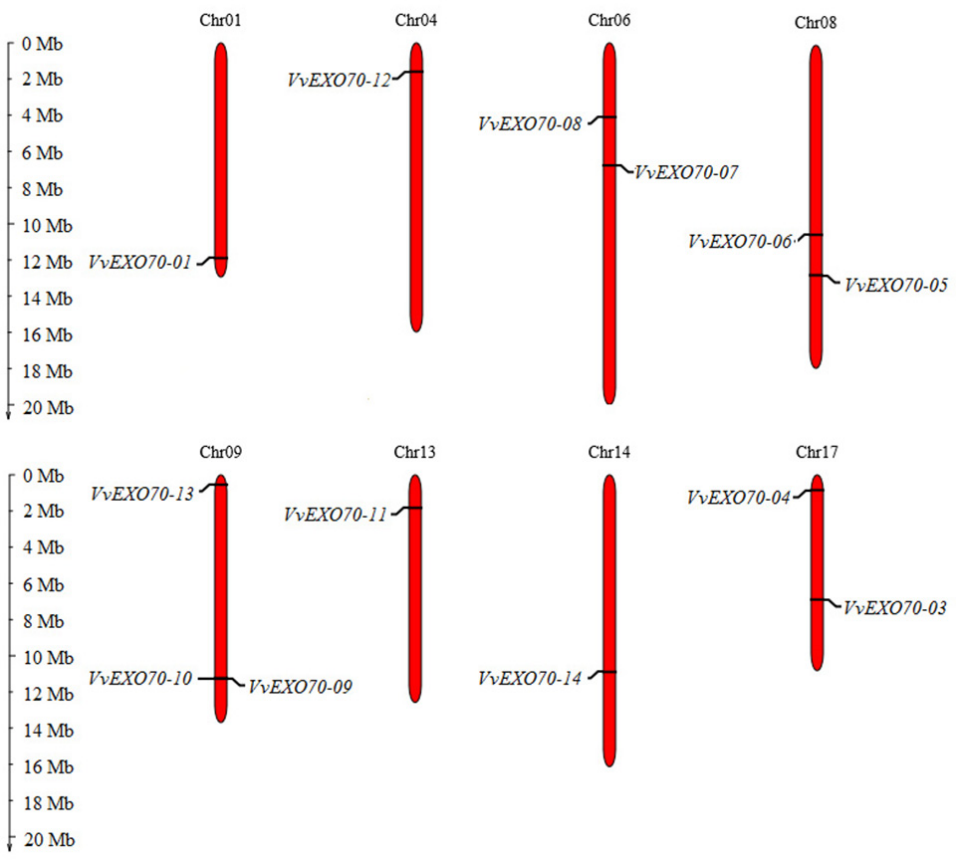
Distribution of VvEXO70 gene family on chromosome. Chromosomal locations of 14 *VvEXO70* genes. The red stripe represents some of the chromosomes in the grape, and the chromosome number is showed at the top of each red stripe.

## Results

### Identification and physicochemical characterization of VvEXO70s

Fourteen *VvEXO70* genes were homologously searched and named as *VvEXO70-01* ∼*VvEXO70-14*. Chromosome localization (Fig. 2) showed that the location of *VvEXO70-02* was unknown and the other 13 genes located on eight chromosomes of grape, named Chr01st, Chr04th, Chr06th, Chr08th, Chr09th, Chr13th, Chr14th and Chr17th. Both *VvEXO70-09* and *VvEXO70-10* were located on the same site of Chr09th and belonged a pair of tandem duplication genes. The physicochemical properties of *VvEXO70s* suggested that the number of amino acids mainly were concentrated in 610∼690 aa, but among them *VvEXO70-13* had 452 aa. In all genes, the number of MW of *VvEXO70-06* (77426.01 kD) was the largest and the minimum is *VvEXO70-07* (51585.27 kD). Also, the number of exons was mainly 1, 2, and 3, but the exons of *VvEXO70-02* and *VvEXO70-05* were 12 and 11, respectively (Table 1).

### Analysis of gene structure and subcellular localization of VvEXO70s

Secondary structural analysis (Table S3) showed that the *VvEXO70* gene family was mainly composed of alpha-helix and random coil, and the proportion of the two was similar, with the proportion of beta-turn, not more than 10%. The tertiary structure showed that *VvEXO70-09*, *VvEXO70-10* and *VvEXO70-12* were similar and greatly different from the rest of the genes (Fig. S1).

Subcellular localization (Table S4) found that most of the EXO70 proteins may be locate in the nucleus, cytoplasm, chloroplasts, mitochondria, and plasma membrane. Except for VvEXO70-08 and VvEXO70-11, other proteins would locate in the cytoplasm and nucleus, among which VvEXO70-05 was possibly the most abundant in the cytoplasm, and VvEXO70-01 and VvEXO70-07 were potentially the richest in the nucleus. VvEXO70-08 scored the highest probability to be present only in the chloroplast, In the mitochondria, VvEXO70s maybe have low content. VvEXO70-01 and VvEXO70-03 only potentially existed in peroxidases. VvEXO70-03 and VvEXO70-04 only possibly located in cytoskeleton. There were six genes would locate in the plasma membrane, among which VvEXO70-11 was possibly the most abundant. VvEXO70-06 and VvEXO70-13 could locate in the extracellular matrix. There was only one gene possibly located in the nuclear and nuclear and plasma membrane respectively, and these were VvEXO70-04 and VvEXO70-05. We predicted that genes locating in different organelles have different physiological functions, such as photosynthesis, growth and development and the like.

**Table 1:** Physicochemical property analysis of VvEXO70 gene family.

Gene	Gene Accession No.	Length of CDS	Length of genomic	Length of amino acid	Exon	Molecular weight	Isoelectric point	Instability index	Aliphatic index	Grand average of hydropathicity
*VvEXO70-01*	GSVIVP00006977001	1920	1965	639	2	72341.22	5.18	53.78	72.18	−0.554
*VvEXO70-02*	GSVIVP00013809001	1953	33699	650	12	73686.77	8.12	45.36	84.03	−0.443
*VvEXO70-03*	GSVIVP00016199001	1932	1932	643	1	73525.06	5.56	46.58	96.30	−0.304
*VvEXO70-04*	GSVIVP00017936001	1974	1974	657	1	74221.00	4.89	47.88	87.88	−0.358
*VvEXO70-05*	GSVIVP00021515001	1947	8150	648	11	73278.55	7.34	44.58	86.59	−0.406
*VvEXO70-06*	GSVIVP00023109001	2067	2085	688	2	77426.01	6.78	57.26	78.43	−0.344
*VvEXO70-07*	GSVIVT00024003001	1363	1733	452	1	51585.27	6.74	49.77	94.49	−0.224
*VvEXO70-08*	GSVIVP00024408001	2067	2085	621	1	70148.30	6.21	53.02	78.68	−0.362
*VvEXO70-09*	GSVIVP00025283001	2052	7574	683	2	77236.89	7.84	41.42	98.78	−0.178
*VvEXO70-10*	GSVIVP00025287001	1971	2007	656	2	74589.93	6.60	40.11	100.93	−0.170
*VvEXO70-11*	GSVIVP00029521001	2025	8680	674	3	76259.83	6.62	44.74	94.33	−0.023
*VvEXO70-12*	GSVIVP00032320001	1899	2004	632	2	71019.17	6.85	42.21	106.65	−0.108
*VvEXO70-13*	GSVIVP00034051001	1836	1836	611	1	69560.89	5.34	50.30	92.13	−0.301
*VvEXO70-14*	GSVIVP00038035001	1959	2491	652	1	73953.32	4.82	51.84	90.90	−0.370


### Phylogenetic evolution, Multiple sequence alignment and motif analysis of VvEXO70s

**Figure 3:**
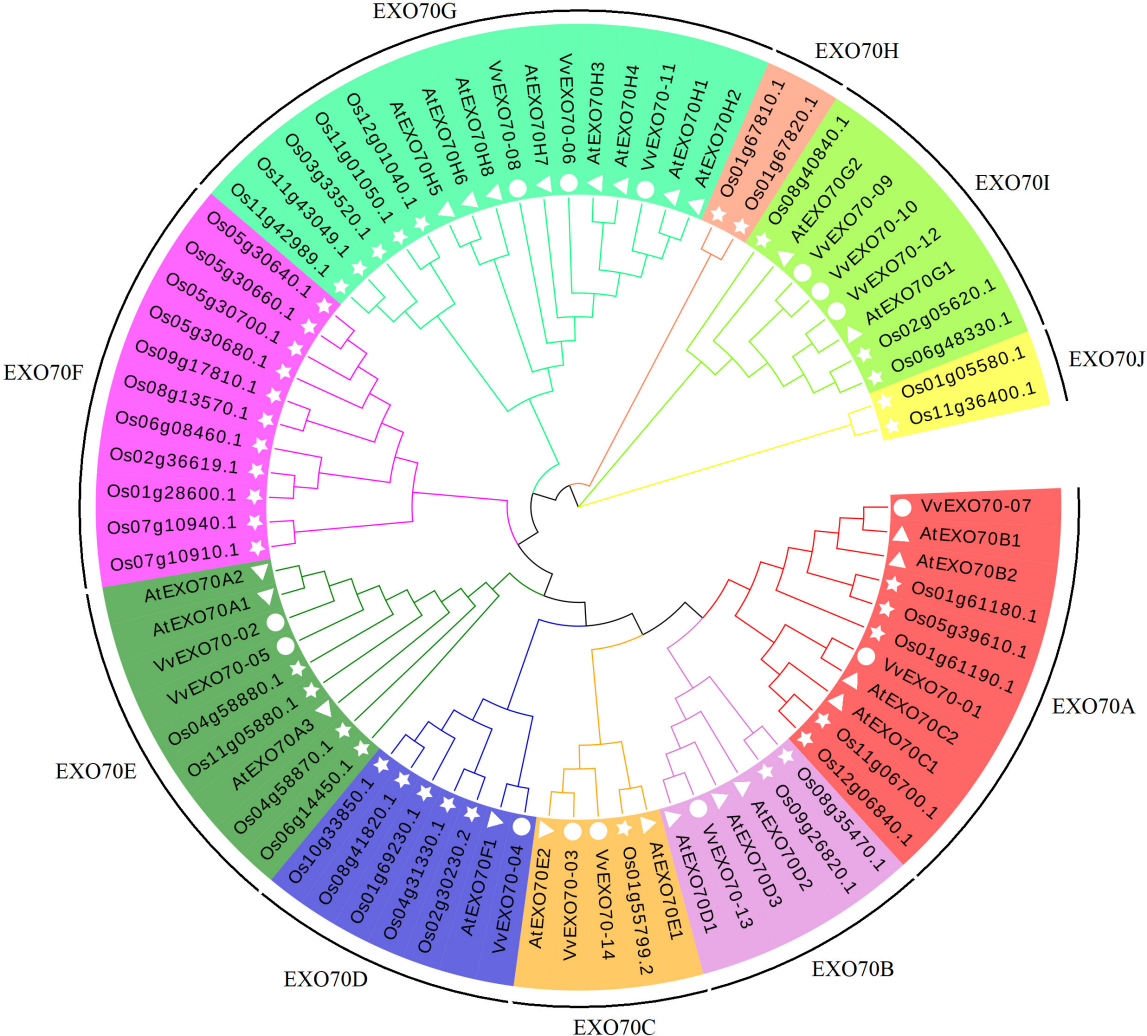
Grape, Arabidopsis thaliana, rice evolution tree of EXO70 gene family. Grape, *A. thaliana*, rice evolution tree of *EXO70* gene family. White circle, triangle, star represent grapes, *A. thaliana*, and rice *EXO70* genes, respectively. Lines and background with different colors represent different subgroups.

**Figure 4:**
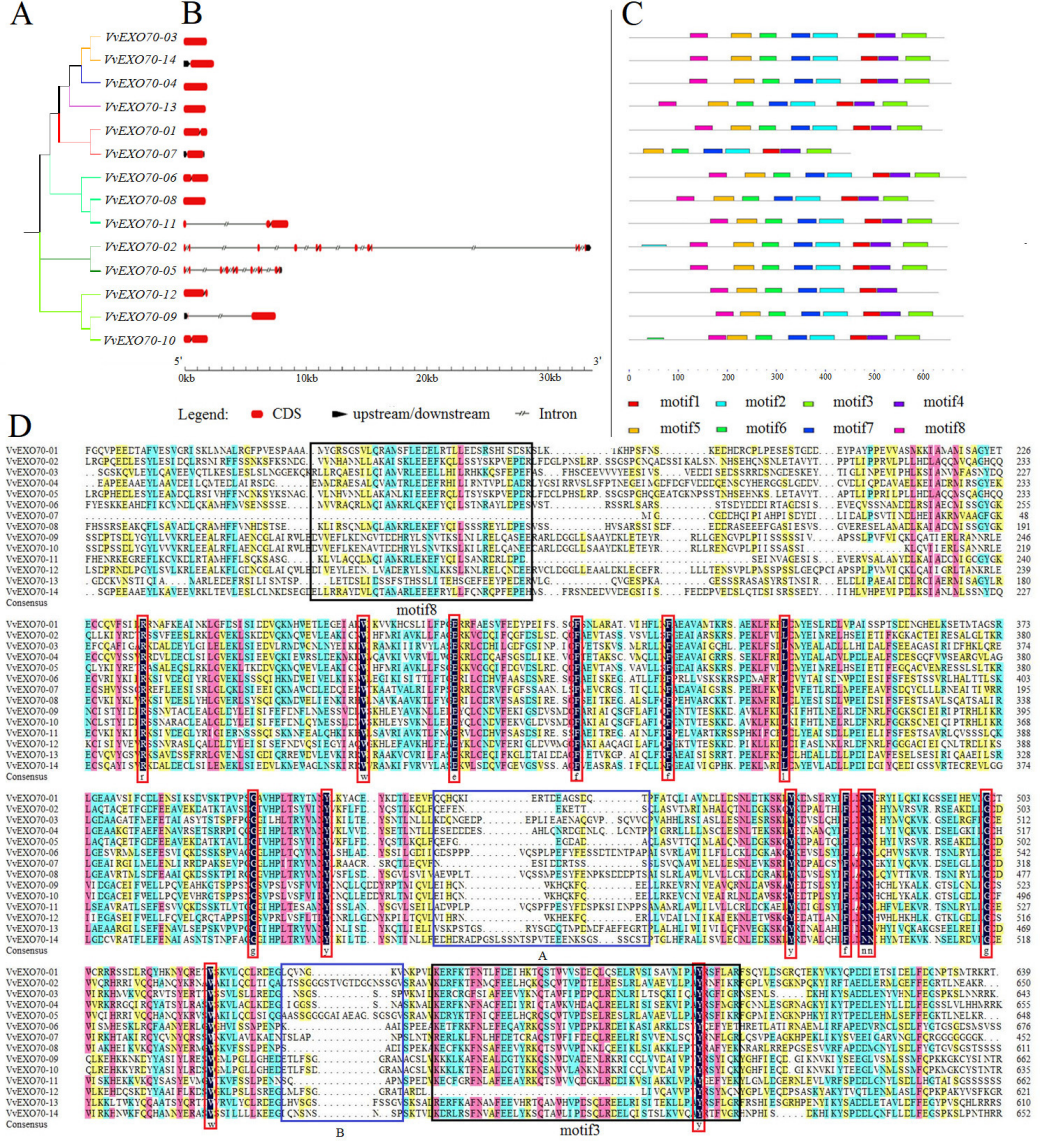
Gene structure, motif and multiple sequence analysis of VvEXO70 gene family. (A) Cluster analysis of *Vv EXO70 s*. (B) Genes structure of *Vv EXO70 s*. The exon, intron and upstream/downstream are represented by red boxes, gray lines and black boxes. (C) Protein motif. Different color represented different motif. (D) Multiple sequence result.

In this study, all 77 EXO70 protein sequences of grape (14), *A. thaliana* (23) and rice (40) were used to construct a phylogenetic tree. Figure 3 showed that they were divided into 10 sub-groups, namely EXO70A ∼J, which consisted 11, 6, 5, 7, 9, 11, 16, 2, 8 and 2 members respectively. Sub-group EXO70A, B, C, D, E, and G contained grape, *A. thaliana* and rice genes. EXO70F, H and J did not contain the *VvEXO70* genes but only rice genes. Meanwhile, Fig. 4A showed that the groups of the separate cluster analysis of *VvEXO70* only had seven ones.

Besides, Fig. 4B showed that all *VvEXO70* genes had different structures. *VvEXO70-03*, *VvEXO70-04*, *VvEXO70-08*, and *VvEXO70-13* had only CDS fragments, no upstream and downstream gene sequences and introns. *VvEXO70-09* and *VvEXO70-14* had the upstream gene sequence. *VvEXO70-02* and *VvEXO70-05* had the downstream gene sequence. *VvEXO70-07* was the only one gene containing upstream and downstream sequences in all genes. *VvEXO70-02* had the longest fragment length that was more than 33 kb. Moreover, cluster analysis of genes with similar structure was found in the same subgroup.

In this experiment, 8 motifs were constructed and named motif1 ∼8. The result (Fig. 4C) showed that *VvEXO70-07* did not have motif8, *VvEXO70-12* had no motif3. This was consistent with the result of multiple sequence alignment (Fig. 4D), which showed that *VvEXO70-12* had no complete motif3 and the motif8 of *VvEXO70-07* was incomplete. The remaining genes contained all motifs. It indicated that the gene family structure was similar and had certain conservatism in evolution.

Protein sequences of the VvEXO70 family were compared and some regions were intercepted (Fig. 4D). It mainly contained the specific structure domain ‘PF03081.15’ with base loci from about 250∼650 aa. Within its domain, it contained 15 highly conserved loci representing by red boxes in the Fig. 4D. At the same time, two highly fractured zones were found in the conservative structure domain, which were named by A and B in it, we speculated that the gene family had some variation during the evolutionary process.

### *cis*-acting element analysis of VvEXO70s

**Figure 5:**
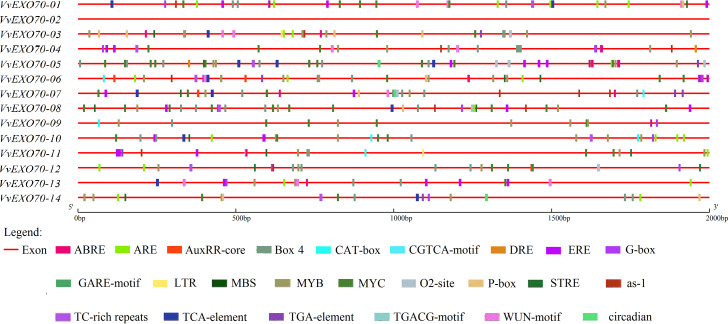
cis-acting elements existed in the 2 kb upstream region of VvEXO70 gene family. The distribution of the 24 *c is*-acting elements in the promoter sequence of 14 *VvEXO70* genes, shown in different colors. ABRE, *cis*-acting element involved in the abscisic acid responsiveness. ARE (anaerobic induction). AuxRR-core (auxin responsiveness). Box-4 (part of a conserved DNA module involved in light responsiveness). CAT-box (meristem expression). CGTCA-motif (MeJA-responsiveness). DRE (Drought). ERE (ETH). G-Box (light responsiveness). GARE-Motif (gibberellin-responsive). LTR (low-temperature responsiveness). MBS (MYB binding site involved in drought-inducibility). MYB (Drought). O2-site (zein metabolism regulation). P-box (gibberellin-responsive). STRE (Pressure). as-1 (SA). TCA-element (SA). TGA-element (auxin-responsiv). TGACG-Motif (MeJA-responsiveness). WUN-motif (callus). Circadian (circadian control).

The analysis of *cis*-acting elements is very important to understand the regulatory mechanism of genes. The online plant-care software tool was used to analyze the codon in the promoter sequences of 2 kb upstream of *VvEXO70* gene, and it was found that the regulatory elements of the *VvEXO70*s were very abundant in number and variety. Main 24 types of hormone and stress related *cis*-acting regulatory elements were identified in the promoters of *VvEXO70* genes (Fig. 5). However, we found nothing about the functional components of *VvEXO70-02* on the online site. Emphasis was placed on the analysis of *cis*-acting elements in *VvEXO70* genes related to grape growth and development, hormones and abiotic stress response. There are mainly the following types.

*cis*-acting element involved in light responsiveness ; Box-4, G-box. *cis*-acting element involved in hormone: CGTCA-motif and TGACG-motif are related to MeJA responsive element. GARE-motif and P-box are related to Gibberellin-responsiveness responsive element. TCA-element and as-1 are related to SA responsive element, as well as Auxin responsive element and Abscisic acid responsive element. In addition, there are *cis*-acting regulatory element involved in circadian control, cell cycle regulation and abiotic stress, including low temperature responsive element, WUN-motif element and anaerobic induction element, MYB binding site involved in drough-induibility and so on. Figure 5 and Table S5 showed that all of *VvEXO70s* except *VvEXO70-02* contained different species numbers of *cis*-acting elements. 13 *VvEXO70* genes contained G-box and Box-4 elements associated with light stress and MYB and MYC elements associated with drought. In addition, there are 28, 30 and 40 elements related to SA, MeJA and ET. The results suggested that the *VvEXO70s* were involved in regulating various hormones and abiotic stresses to cope with various adverse environments.

### Analysis of collinearity and selective pressure between grape and *A. thaliana* EXO70 genes

**Figure 6:**
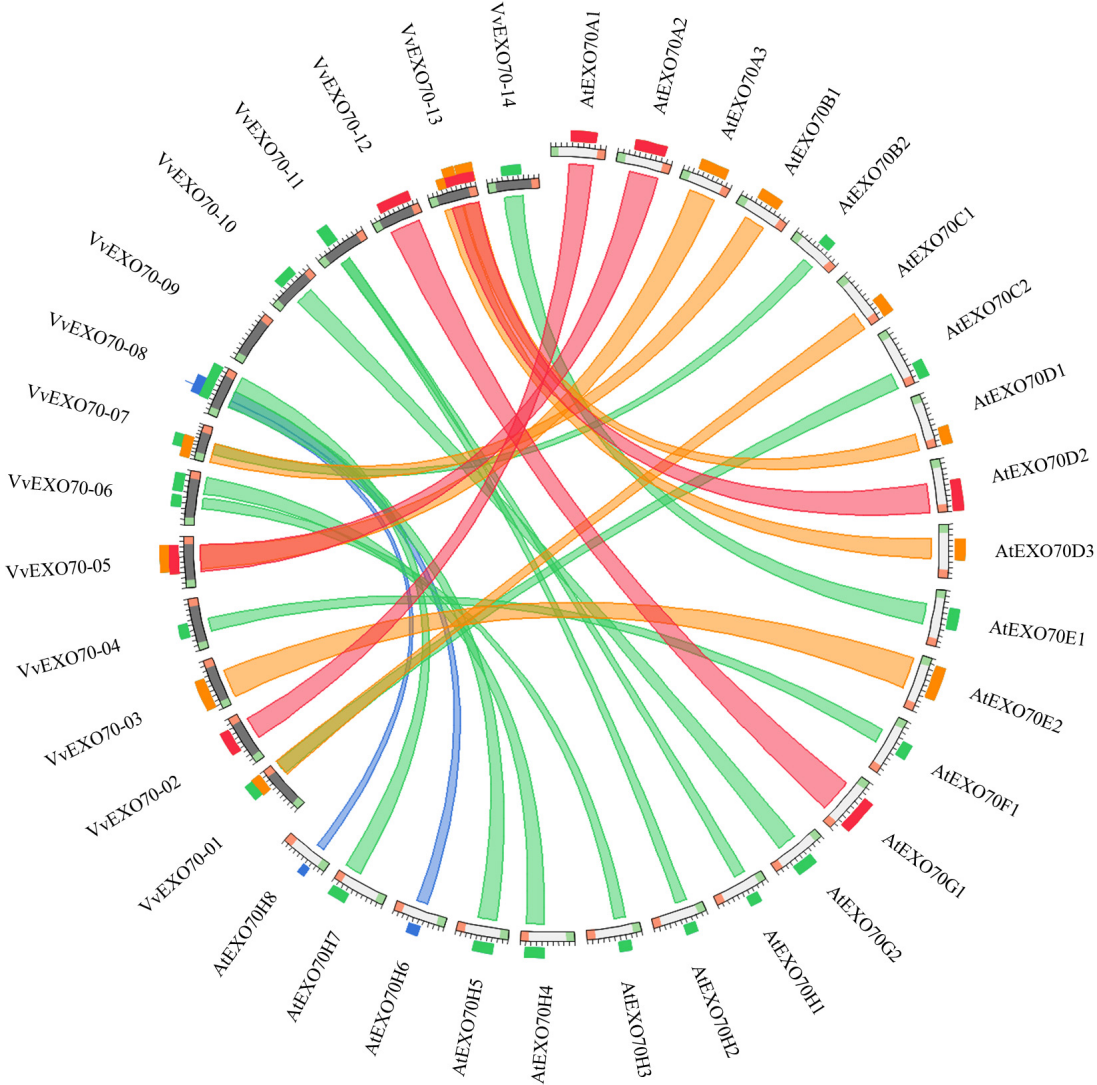
Collinearity analysis of the EXO70 gene family between grape and *Arabidopsis thaliana*. The color of the strips represents the extent of similarity and homology among the genes. Blue indicates the lowest similarity, followed by green, orange and red, indicating the increasing similarity.

The online software was used to obtain the collinearity relationship of *EXO70* genes between grape and *A. thaliana* (Fig. 6). It was found that there were 23 pairs of collinearity genes between grape and *A. thaliana*. Red indicated the largest similarity, followed by orange, green and blue, indicating the decreasing similarity. For example, in the red ribbon, *VvEXO70-02* and *AtEXO70A1*, *VvEXO70-05* and *AtEXO70A2*, *VvEXO70-12* and *AtEXO70G1*, *VvEXO70-13* and *AtEXO70D2* had the strongest collinearity. There were 6 pairs of genes with collinear relationship on orange degree. The next weaker collinearity was found in 11 pairs of genes. However, the two weakest pairs were *VvEXO70-08* and *AtEXO70H6*, *VvEXO70-08* and *AtEXO70H8*. In addition, the collinear relationship between *VvEXO70* and *AtEXO70* was one to many, such as the collinear relationships between *VvEXO70-08* and *AtEXO70H5*, *AtEXO70H6*, *AtEXO70H7* and *AtEXO70H8* were be in varying degrees.

In the process of gene evolution, mutations include synonymous mutation and non-synonymous mutation, and 3 important values, named the synonymous mutation frequency (d_S_) and the non-synonymous mutation frequency (d_N_) and the ratio of d_N_/d_S_. The value of d_N_/d_S_ plays an important role in gene selection and evolution. When d_N_/d_S_ >1 is the positive selection, d_N_/d_S_ = 1 is the neutral selection, 0 <  d_N_/d_S_ < 1 is purifying selection (Yang & Bielawski, 2000;Yadav et al., 2015). To further explore the evolutionary pattern of this gene family, we found that the values of d_N_/d_S_ of all 23 duplicated gene pairs were less than 1 between grape and *A. thaliana*, indicating that the evolution pattern was purifying selection, which played a crucial role in keeping the number of *EXO70* in grape and could help to maintain the basic function of this gene (Table 2).

**Table 2:** Selective pressure analysis of *VvEXO70* gene family.

Ribbon color	A pair of genes	S	N	d_S_	d_N_	d_N_/d_S_
Red	*VvEXO70-02*∼*AtEXO70A1*	466.2	1,447.8	1.4522	0.0966	0.0665
	*VvEXO70-05*∼*AtEXO70A2*	463.9	1,429.1	1.6586	0.1357	0.0818
	*VvEXO70-12*∼*AtEXO70G1*	444.8	1,451.2	2.1721	0.1488	0.0685
	*VvEXO70-13*∼*AtEXO70D2*	449.1	1,362.9	4.5850	0.2343	0.0511
Orange	*VvEXO70-01*∼*AtEXO70C1*	446.5	1,407.5	49.3070	0.3641	0.0074
	*VvEXO70-03*∼*AtEXO70E2*	495.2	1,406.8	2.8294	0.4196	0.1483
	*VvEXO70-05*∼*AtEXO70A3*	146.4	1,323.6	2.1672	0.3704	0.1709
	*VvEXO70-07*∼*AtEXO70B1*	348.6	1,001.4	2.5887	0.1652	0.0638
	*VvEXO70-13*∼*AtEXO70D1*	452.6	1,362.4	3.6151	0.2335	0.0646
	*VvEXO70-13*∼*AtEXO70D3*	450.1	1,355.9	2.8325	0.2158	0.0762
Green	*VvEXO70-01*∼*AtEXO70C2*	421.2	1,483.8	6.1042	0.3901	0.0639
	*VvEXO70-04*∼*AtEXO70F1*	415.3	1,516.7	5.6402	0.2153	0.0382
	*VvEXO70-06*∼*AtEXO70H3*	470.6	1,380.4	3.1632	0.4221	0.1334
	*VvEXO70-06*∼*AtEXO70H4*	480.9	1,382.1	39.8698	0.3318	0.0083
	*VvEXO70-07*∼*AtEXO70B2*	369.1	950.9	3.8067	033113	0.0818
	*VvEXO70-08*∼*AtEXO70H5*	446.4	1,323.6	13.2657	0.4503	0.0339
	*VvEXO70-08*∼*AtEXO70H7*	463.4	1,354.6	5.5653	0.3917	0.0704
	*VvEXO70-10*∼*AtEXO70G2*	489.8	1,448.2	4.1836	0.5181	0.1238
	*VvEXO70-11*∼*AtEXO70H1*	450.4	1,343.6	4.0569	0.3224	0.0795
	*VvEXO70-11*∼*AtEXO70H2*	468.3	1,331.7	2.6233	0.3326	0.1268
	*VvEXO70-14*∼*AtEXO70E1*	481.7	1,438.3	7.4183	0.3943	0.0531
Blue	*VvEXO70-08*∼*AtEXO70H6*	440.3	1,335.7	17.8262	0.4608	0.0258
	*VvEXO70-08*∼*AtEXO70H8*	444.5	1,256.5	34.1171	0.4399	0.0129

**Notes.**

‘S’ represents the numbers of synonymous, ‘N’ represents non-synonymous, ‘dS’ represents the synonymous mutation frequency and dN represents the non-synonymous mutation frequency and the ratio of dN/dS.

### Analysis of base number and codon usage bias of VvEXO70s

RSCU is the ratio of the actual value used by the codon to the theoretical value used by that (Sharp & Li, 1986; Sharp & Li, 1987). The theoretical value is the value with the same frequency of codon, that is, there is no codon bias. If RSCU=1, the use of codon has no preference. If RSCU>1, it indicates that the codon is used more frequently than other synonymous codons, named a high-frequency codon. If RSCU<1, the codon is used less frequently than other codons (Lin et al., 2002; Li et al., 2016). RSCU values results (Table S6) of *VvEXO70s* codon showed that 14 *VvEXO70* genes contained a total of 8871 codons (excluding the termination codon). Among them, there were 5984 codons’ RSCU>1, 2620 codons ending in A/U, and 3364 codons ending in C/G, accounting for 43.78% and 52.22% of the total codons of RSCU>1, respectively. This suggested that the codon ending in C/G was the preferred codon of the *EXO70* gene family. Among all codons, the RSCU value of AGG codon of *VvEXO70-10* gene was the highest (3.18). Some RSCU values of codons ending in G/C differed from others, for example, RSCU values of UUC codons of *VvEXO70-01*, *VvEXO70-04*, *VvEXO70-06*, *VvEXO70-07* and *VvEXO70-08* were greater than 1, while RSCU values of other UUC codons were less than 1.

Analysis of the codons usage parameters of *VvEXO70s* is shown in Table S7. ENC, CAI and CBI values were used to predict gene expression levels. The ENC value of the effective codon ranges from 20 to 61, and the smaller the value, the stronger the applicability of the gene codon (Gupta, Bhattacharyya & Ghosh, 2004; Satapathy et al., 2017). When the use bias reaches the maximum degree, the ENC value is 20; The smaller the usage bias, the closer to 61 (Wright, 1990). The higher the codon preference was, the higher the gene expression level was, and the ENC value was negatively correlated with CAI and CBI values. Low expression gene has low preference, and its CAI and CBI values are small, which will be affected by the amino acid composition and gene length of the gene (Wright, 1990; Marashi & Najafabadi, 2004). Furthermore, we found the average of ENC of *VvEXO70s* was 54.84, the minimum value was 53.2 (*VvEXO70-10*), and the maximum value was 59.07 (*VvEXO70-07*), indicating that the codon preference of this gene family was weak and the usage degree of deviation from random selection was relatively consistent. In general, CAI and CBI values were positively correlated, with values ranging from 0 to 1. Genes, with higher expression, had higher CAI and CBI values. The closer to 1, the higher the value, and the stronger the codon preference (Wu et al., 2007). In addition, the CAI mean of *VvEXO70s* was 0.203 and the CBI mean was −0.031. The above results indicated that *VvEXO70* gene family had weak codon preference. Among them, CAI and CBI values of *VvEXO70-01* were the maximum values, which were 0.267 and 0.102 respectively. In addition, the Fop value of *VvEXO70-01* gene was the maximum value among all members, indicating that the gene contained the codon with the highest frequency of usage. Further analysis of GC contents of 14 *VvEXO70* genes and GC, A, T, C and G in the third place of the codon showed that the average G3s content was 36.97% and the average C3s content was 29.29%. The average content of T3s and A3s was 34.94% and 26.71%, respectively. The average GC content was 46.80%, and the average GC3s content was 50.60%. This indicated that the base selection of the third position of the code of *VvEXO70s* had a weak C/G preference, and the codon ending in C/G was preferred.

Correlation coefficient between parameters of *VvEXO70s* codon usability (Table S8) showed that CAI values, CBI values and FOP values were extremely positively correlated with each other (*p* < 0.01). C3s values were extremely positively correlated with CBI, Fop, GC3s and GC values (*p* < 0.01). GC3s values were extremely positively correlated with GC values (*p* < 0.01). L_sym and L_aa values were extremely positively correlated (*p* < 0.01). GC values were positively correlated with CBI and Fop values (*p* < 0.05). T3s values were extremely negatively correlated with C3s, CBI, Fop, GC3s and GC values (*p* < 0.01). A3s values were extremely negatively correlated with C3s and GC3s values (*p* < 0.01), and negatively correlated with GC values (*p* < 0.05). ENC values were negatively correlated with L_sym and L_aa values (*p* < 0.05). It showed that the base content in the third position of the synonymous codon had a high influence on the gene expression level and codon preference (Zhang et al., 2011).

### Microarray of VvEXO70s

**Figure 7:**
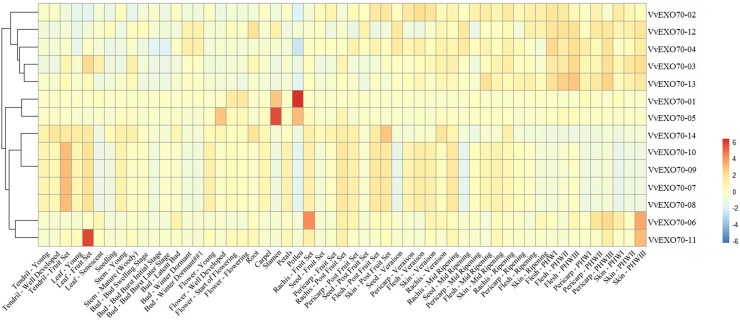
The expression pattern of VvEXO70 gene family in different tissues and organs. The band indicates the level of gene expression.

Expression patterns of all *VvEXO70* from transcriptomic data from grape organs and tissues at different stages of development and under abiotic stress were studied (Fig. 7). The data were mainly from a total of 54 grapevine samples, covering most of the grape organs at different stages, were collected. Three biological replicates were taken for each sample, resulting in a total of 162 observations. The collected plant organs were: bud, inflorescence, tendril, leaf, stem, root, developing berry, withering berry, seed, rachis, anther, carpel, petal, pollen, and seedling. The results showed that most genes are expressed to varying degrees in tendrils, leaves, seeds, buds, roots and stems. Among them, *VvEXO70-01* was mainly found in pollen and stamens, with the highest expression in pollen. *VvEXO70-02* was mainly found in pericarp-veraison, seed-veraison and skin-veraison. *VvEXO70-04* was detected in bud-winter dormant, pericarp-veraison, seed-veraison and flesh-PHWI, and its expression level was relatively higher than that of other genes. In all the tissues, *VvEXO70-05* had the highest content in stamens, followed by pollen and flower-well developed. The expression of *VvEXO70-11* was the highest in the leaf-fruit set. *VvEXO70-13* had the highest expression in flesh-PHWIII. However, *VvEXO70-04* did not expressed in pollen, bud, leaf, seedling and skin-ripening. *VvEXO70-14* did not expressed in skin-rippen, flesh and pericarp. All *VvEXO70-07*, *VvEXO70-08*, *VvEXO70-09* and *VvEXO70-10* didn’t expressed in leaf-sensecent, seedling, bud, carpel, pollen, seed, flesh-ripening, pericarp and skin-PHWI, -PHWII, -PHWIII.

**Figure 8:**
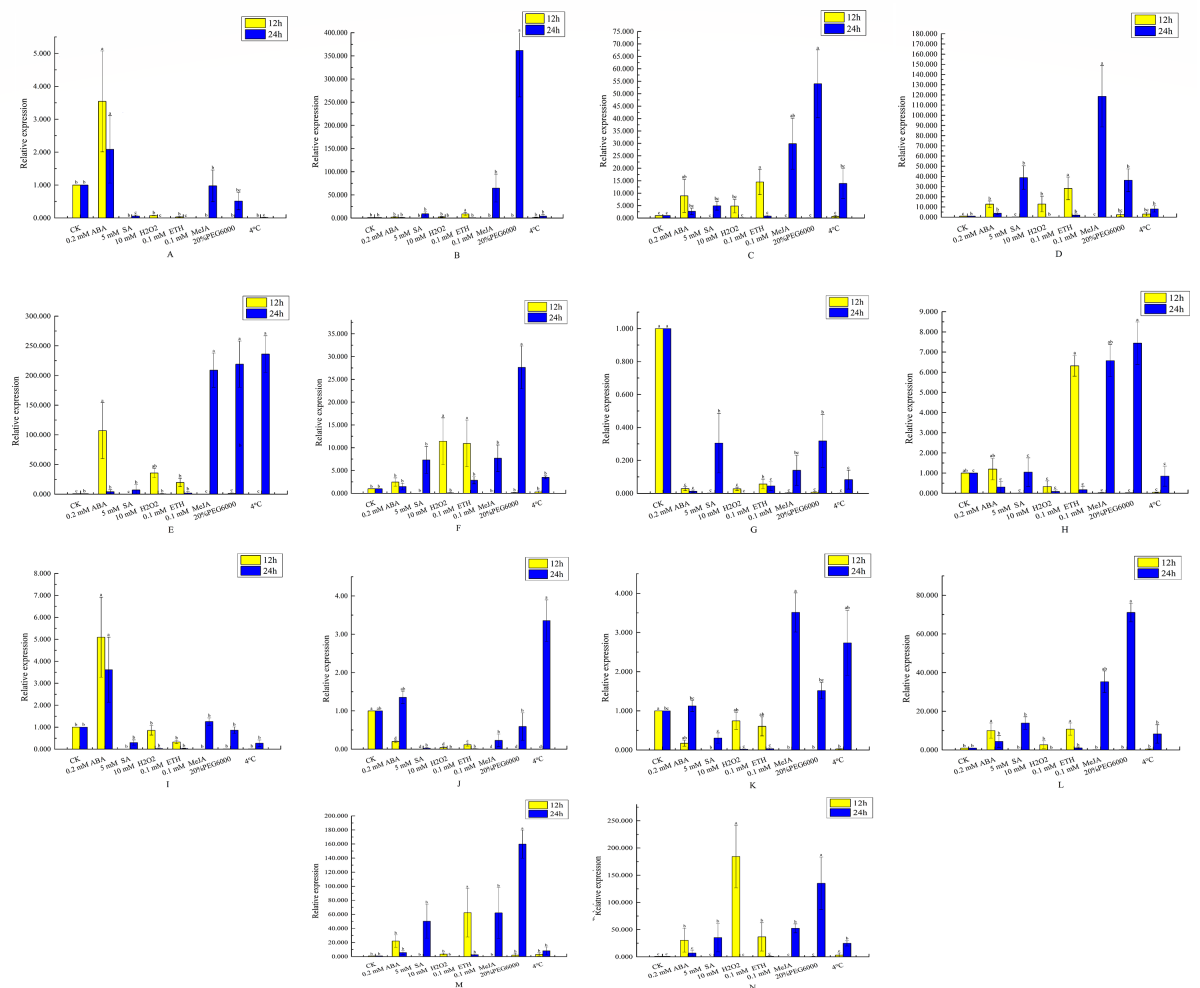
Analysis of qRT-PCR expression results of VvEXO70 gene family. 14 *VvEXO70* genes were analyzed by qRT-PCR which were used to assess *VvEXO70* relative expression levels in leaves sampled at 12 h and 24 h. A∼ N represent *VvEXO70 -01*∼*VvEXO70 -14* gene, respectively. Yellow and blue represent the relative expression for 12 h and 24 h, respectively.

### qRT-PCR analysis of VvEXO70s

This study further verified and analyzed the relative expression characteristics of *VvEXO70* s. Figure 8 showed that there were significant differences in the relative expression levels of part of genes when all *VvEXO70s* genes were induced by different hormones and abiotic stresses about 12 h and 24 h. The relative expression levels of all genes in 5 mM SA, 0.1 mM MeJA, 20% PEG6000 and 4 °C for 24 h were all higher than that for 12 h. Compared to the CK, the relative expression quantity of *VvEXO70-01, 02*, *03*, *04*, *05*, *06*, *09*, *12*, *13*, and *14* up-regulated under 2 mM ABA treatment about 12 h and 24 h. On the contrary *VvEXO70-07*’s relative expression was significantly down-regulated. After 20% PEG6000 treatment for 24 h, the relative expression levels of *VvEXO70-02* and *VvEXO70-05* significantly up-regulate and were 361 and 219 times higher than that of CK respectively. When the leaves were subjected to 4 °C, *VvEXO70-05* had the highest relative expression about 24 h. With 2 mM ABA about 12 h, the relative expression of *VvEXO70-05* was highest. *VvEXO70-13’s* relative expression with 5 mM SA about 24 h was the highest. Most of the genes were up-regulated after 0.1 mM MeJA, especially *VvEXO70-05’s* at 24 h. Under 7 mM H_2_O_2_, all genes’ expression for 12 h were higher than 24 h, among which *VvEXO70-02*, *03*, *04*, *05*, *06* and *14* significantly up-regulated. This result showed that many *VvEXO70* genes were likely to play critical roles in the abiotic and hormonal stress signaling transduction pathways.

## Discussion

Exocyst belongs to one of ancient families of the eukaryotic tetraploid system, which is an octameric vesicle mesenteric complex acting upstream of SNARE mediated fusion of extracellular vesicles with the plasma membrane (Whyte & Munro, 2002; Zhang et al., 2010). EXO70, a subunit of the exocyst, together with SEC3 depends on PIP2 in the cell membrane localization. Under the synergistic action of these three proteins, a cell membrane target polarity site is established and then interacted with other 6 exocrine complex components to tether exocrine vesicles to the cell membrane (He et al., 2007; Zhang et al., 2008; Li et al., 2010). This study has found that *VvEXO70* gene family consisted of 7 subfamilies (Fig. 4A), which could trace to three ancient *EXO70* genes existing in common ancestor of mosses and vascular plants, named *EXO70.1*, *EXO70.2* and *EXO70.3*. These genes were independently replicated in mosses, lycopodium, and angiosperms lineages (Synek et al., 2006; Chong et al., 2010; Fatima et al., 2012).

In this experiment, a total of 14 *VvEXO70* genes were obtained by bioinformatics and domain comparison, which were named *VvEXO70-01 ∼VvEXO70-14*. Grape, *A. thaliana*, rice protein sequence were used to construct a phylogenetic tree divided into EXO70A ∼J (Fig. 3), in which EXO70F, H and J did not contain *VvEXO70* gene, but only *OsEXO70* gene, which was the same as previous research groups (Fatima et al., 2012; Yang et al., 2015a). There were differences in the distribution of exons in the same subgroup, which was similar to the results of Yang et al. (2015b) in cabbage and Chinese cabbage and this suggested that *EXO70* genes may have some similarities in the evolution of different species. Multiple sequence alignment (Fig. 4D) and motif analysis (Fig. 4C) showed that VvEXO70 proteins contained a complete pfam03081 domain, which was composed of most of the *α*-helix and was a key factor in determining the function of EXO70 proteins. Dellago et al. (2011) found that EXO70 could participate in pre-mRNA clipping, and all known EXO70 interacted with other proteins. For this, the conservative pfam03081 may be a necessary domain for interaction with other proteins, but its specific function has not been further studied (Yang et al., 2015a) Besides, the domain had two highly fractured bases, A and B (Fig. 4D). The remaining genes contained all motifs except for *VvEXO70-07* and *VvEXO70-12*, so we predicted that this gene family could have high structural similarity and conservatism in the evolutionary process, and certain variation. It was found that the C-terminal of *EXO70* gene was the most conserved structural domain in the whole molecule, which contained a large number of basic residues such as Arg and Lys. It was close to the plasma membrane and further associated with Rho3p through the interaction between the clustered alkaline residues and Rho3p (Dong et al., 2005; Hamburger et al., 2006).

Understanding the protein’s subcellular location information (Table S4) may provide us with the necessary help to infer the biological function of the protein, *VvEXO70s* woud mainly locate in nucleus, cytoplasm, chloroplast, mitochondria and plasma membrane so it was speculated to be related to plant cytoplasmic secretion, photosynthesis, respiratory action and cell growth and development. Plant tissue expression specificity (Fig. 7) found that most of *VvEXO70* gene were expressed to varying degrees in pollen, tendrils, leaves, seeds, buds, roots and stems. Among them, *VvEXO70-01* was mainly found in pollen and stamens. *AtEXO70A1* expresses in all plant tissues by gene chip and qRT-PCR (Synek et al., 2006; Chong et al., 2010). Moreover, Li et al. (2010) also found that *AtEXO70* gene mainly expresses in tissues with high exocrine activity, such as elongated cells at growth points and developed xylem molecules, while the expression was low in other tissues. Yang & Bielawski (2000) detected *BoEXO70A1* expression in stem, leaf, petal, anther, stigma, style and ovary by the Northern method, indicating that it may be a constitutive expression gene, but it still needs to be further detected. These indicated that the *EXO70* genes family may be related to the tip growth and elongation development of plants.

The collinear relationship (Fig. 6) between grape and *A. thaliana EXO70* genes was analyzed, and 23 pairs of collinear genes were found between grape and *A. thaliana*. Also, the d_N_/d_S_ values of these gene replication pairs were all less than 1, indicating that they had been purified and selected to predict their high conservatism in the evolution process (Table 2). The analysis of codon bias (Table S7) showed that the codon bias of the members of the gene family was weak and the degree of deviation from random selection was consistent. However, Yang et al. (2015a) and Yang et al. (2015b) found that the codon bias of cabbage and chinese cabbage was almost identical, while that of *A. thaliana* and rice was significantly different. Besides, according to the correlation of each parameter, the base content in the third position of the synonymous codon has a high influence on the gene expression level and codon preference, it was similar to Zhang et al,. found in 2011. This suggested that the result of using codon preference in the evolutionary process to predict the genomic location of EXO70 for unknown proteins in different species may be different.

When plants are under stresses such as low temperature, drought and hormone, these stress signals will be converted into signal factors, activate the *cis*-acting elements of transcription factor binding genes and stimulate the expression of related genes and responding to stresses (Shinozaki & Shinozaki, 1997; Teixeira et al., 2004; Hadiarto & Tran, 2011). The 2 kb upstream sequences of *VvEXO70s* (Fig. 5) found that VvEXO70s contained various *cis*-acting elements such as G-box and Box-4 elements related to light stress, and MYB and MYC elements related to drought presented in all genes, suggesting that this gene family had certain drought resistance. qRT-PCR (Fig. 8) suggested that *VvEXO70s’* relative expression were higher than CK with 20% PEG6000 treatment for 24 h, indicating that most of *VvEXO70s* had drought resistance, which was similar to the results of *cis*-acting element. In addition, the *VvEXO70s* also contained many other different *cis*-acting elements, such as 28, 30, and 40 components related to SA, MeJA, and ET, respectively. Pieterse et al. (2009) also find that plant hormones SA, MeJA and ET were important regulatory factors in plant resistance signal transduction pathways. It is speculated that these *cis*-acting elements could have a great role in plant growth and resistance. qRT-PCR (Fig. 8) found that many *VvEXO70* genes had different responses to stresses. For example, all genes’ relative expression in 5 mM SA, 0.1 mM MeJA, 20% PEG6000 and 4 °C for 24 h were all higher than that for 12 h. After 20% PEG6000 treatment for 24 h, the relative expression levels of *VvEXO70-02* and *VvEXO70-05* significantly up-regulate and were 361 and 219 times higher than that of CK respectively. *VvEXO70-13*’s relative expression under 5 mM SA for 24 h was the highest. Most of genes up-regulatedly expressed with 0.1 mM MeJA, especially *VvEXO70-05*’s at 24 h. The relative expression level of all genes with 7 mM H_2_O_2_ for 12 h were higher than 24 h, among which *VvEXO70-02*, *03*, *04*, *05*, *06* and *14* significantly up-regulated. *VvEXO70-05* had the highest relative expression with 4 °C for 24 h. With 2 mM ABA treatment for 12 h, the relative expression of *VvEXO70-05* was the highest and of *VvEXO70-07* significantly down-regulate compared to the CK, This showed that many *VvEXO70* genes were likely to play critical roles in the abiotic and hormonal stress signaling transduction pathways.

## Conclusion

*VvEXO70* gene family consists of 14 members, and most genes expressed to varying degrees in tendrils, leaves, seeds, buds, roots and stems. In addition, qRT-PCR analysis showed *VvEXO70-02*, *VvEXO70-03*, *VvEXO70-04*, *VvEXO70-05*, *VvEXO70-13* and *VvEXO70-14* may have certain function in resisting abiotic and hormone stresses*.* This provides ideas for further exploring the function of *VvEXO70s* and breeding work of grape in the later stage.

##  Supplemental Information

10.7717/peerj.11176/supp-1 Supplemental Information 1 The tertiary structure of *VvEXO70* gene family

10.7717/peerj.11176/supp-2 Supplemental Information 2 Numbers of genes encoding *EXO70* in plant genomes

10.7717/peerj.11176/supp-3 Supplemental Information 3 qRT-PCR primers for expression on analysis of *VvEXO70* gene family

10.7717/peerj.11176/supp-4 Supplemental Information 4 The secondary structure of *Vv EXO70* protein sequences

10.7717/peerj.11176/supp-5 Supplemental Information 5 Subcellular location prediction of *Vv EXO 70* gene family

10.7717/peerj.11176/supp-6 Supplemental Information 6 *c is*-acting elements existed in the 2 kb upstream region of *VvEXO70* gene family

10.7717/peerj.11176/supp-7 Supplemental Information 7 The Codon number and RSCU value of *VvEXO70* gene family

10.7717/peerj.11176/supp-8 Supplemental Information 8 Codon usage feature of *VvEXO70* gene family

10.7717/peerj.11176/supp-9 Supplemental Information 9 Codon usage correlation analysis of *VvEXO70* gene family

10.7717/peerj.11176/supp-10 Supplemental Information 10 Original qRT-PCR data

10.7717/peerj.11176/supp-11 Supplemental Information 11 The amino acid sequence of grape, Arabidopsis thaliana, and rice

## Additional Information and Declarations

### Data Availability

The following information was supplied regarding data availability: Original qRT-PCR data and grape, Arabidopsis thaliana, and rice amino acid sequences are available in the Supplementary Files.

## References

[ref-1] Bu C, Gao S, Dong SJ, Zhao L, Li SP (2019). Advances in the study of exictus in response to plant and biological stress. Life Sciences.

[ref-2] Cai HQ, Reinisch K, Ferro-Novick S (2007). Coats, tethers, Rabs, and SNAREs work together to mediate the intracellular destination of a transport vesicle. Development Cell.

[ref-3] Chen D, Latham J, Zhao H, Bisoffi M, Farelli J, Dunaway-Mariano D (2012). Human brown fat inducible thioesterase variant 2 cellular localization and catalytic function. Biochemistry.

[ref-4] Chong YT, Gidda SK, Sanford C, Parkinson J, Mullen RT, Goring DR (2010). Characterization of the *Arabidopsis thaliana* exocyst complex gene families by phylogenetic, expression profiling, and subcellular localization studies. New Phytologist.

[ref-5] Dellago H, Löscher M, Ajuh P, Ryder U, Kaisermayer C, Grillari-Voglauer R, Fortschegger K, Gross S, Gstraunthaler A, Borth N, Eisenhaber F, Lamond AI, Grillari J (2011). Exo70, a subunit of the exocyst complex, interacts with SNEV (hPrp19/ hPso4) and is involved in pre-mRNA splicing. Biochemical Journal.

[ref-6] Dong G, Hutagalung AH, Fu CM, Novick P (2005). The structures of exocyst subunit Exo70p and the Exo84p C—terminal domains reveal a comm on motif. Nature Structural & Molecular Biology.

[ref-7] Elias M, Drdova E, Ziak D, Bavlnka B, Hala M, Cvrckova F, Soukupova H, Zarsky V (2003). The exocyst complex in plants. Cell Biology International.

[ref-8] Fatima C, Michal G, Radek B, Michal H, Ivan K, Anamika R, Viktor Z (2012). Evolution of the land plant exocyst complexes. Frontiers In Plant Science.

[ref-9] Finger FP, Hughes TE, Novick P (1998). Sec3p is a spatial landmark for polarized secretion in budding yeast. Cell.

[ref-10] Goldman N, Yang Z (1994). A codon-based model of nucleotide substitution for protein-coding DNA sequences. Molecular Biology and Evolution.

[ref-11] Gu YN, Zavaliev R, Dong ZN (2017). Membrane trafficking in plant immunity. Molecular Plant.

[ref-12] Guo W, Grant A, Novick P (1999). Exo84p is an exocyst protein essential for secretion. The Journal of Biological Chemisitry.

[ref-13] Gupta SK, Bhattacharyya TK, Ghosh TC (2004). Synonymous codon usage in lactococcus lactis: mutational bias versus translational selection. Journal of Biomolecular Structure and Dynamics.

[ref-14] Hadiarto T, Tran LS (2011). Progress studies of drought-responsive genes in rice. Plant Cell Reports.

[ref-15] Hamburger ZA, Hamburger AE, West AP, Weis WI (2006). Crystal structure of the & cerevisiae exocyst component Exo70p. Journal of Molecular Biology.

[ref-16] He B, Xi F, Zhang X, Zhang J, Guo W (2007). Exo70 interacts with phospholipids and mediates the targeting of the exocyst to the plasma membrane. EMBO Journal.

[ref-17] Juraj S, Přemysl P, Jiří Š, Nemanja V, Viktor Ž, Martin P (2017). Analysis of Exocyst subunit EXO70 family reveals distinct membrane polar domains in tobacco pollen tubes. Plant Physiology.

[ref-18] Jurgens G, Geldner N (2002). Protein secretion in plants: from the trans-Golgi network to the outer space. Traffic.

[ref-19] Li N, Li YY, Zheng CC, Huang JG, Zhang SZ (2016). Genome-wide comparative analysis of the codon usage patterns in plants. Genes Genom.

[ref-20] Li SP, Van OsG MA, Ren SC, Yu DL, Ketelaar T, Emons AMC, Liu CM (2010). Expression and functional analyses of EXO70 genes in *Arabidopsis* implicate their roles in regulating cell type-specific exocytosis. Plant Physiology.

[ref-21] Lin T, Ni Z, Shen M, Chen L (2002). High-frequency codon analysis and its application in codon analysis of tobacco. Journal of Xiamen University.

[ref-22] Liu DM, Li X, Shen D, Novick P (2018). Two subunits of the exocyst, Sec3p and Exo70p, can function exclusively on the plasma membrane. Molecular Biology of the Cell.

[ref-23] Ma WQ, Wang Y, Yao XM, Xu ZJ, An LG, Yin M (2016). The role of Exo70 in vascular smooth muscle cell migration. Cellular & Molecular Biology Letters.

[ref-24] Marashi SA, Najafabadi HS (2004). How reliable readjustment is: correspondence regarding A. fuglsang, The ‘effective number of codons’revisited. Biochemical and Biophysical Research Communications.

[ref-25] Mathieson EM, Suda Y, Nickas M, Snydsman B, Davis TN, Muller EGD, Neiman AM (2010). Vesicle docking to the spindle pole body is necessary to recruit the exocyst during membrane formation in Saccharomyces cerevisiae. Molecular Cell Biology.

[ref-26] Moore BA, Robinson HH, Xu Z (2007). The crystal structure of mouse Exo70 reveals unique features of the mammalian exocyst. Molecular Biology.

[ref-27] Pieterse CM, Leon-Reyes A, Van der Ent S, Van Wees SC (2009). Networking by small-molecule hormones in plant immunity. Nature Chemical Biology.

[ref-28] Ren J, Guo W (2012). ERK1/2 regulate exocytosis through direct phosphorylation of the exocyst component Exo70. Development Cell.

[ref-29] Samuel MA, Chong YT, Haasen KE, Aldea-Brydges MG, Stone SL, Goring DR (2009). Cellular pathways regulating responses to compatible and selfincompatible pollen in Brassica and *Arabidopsis* stigmas intersect at Exo70A1, a putative component of the exocyst complex. The Plant Cell.

[ref-30] Satapathy SS, Sahoo AK, Ray SK, Ghosh TC (2017). Codon degeneracy and amino acid abundance influence the measure of codon usage bias: improved Nc ( Nc ) and ENCprime ( N′c ) measures. Genes to Cell.

[ref-31] Sharp PM, Li WH (1986). An evolutionary perspective on synonymous codon usage in unicellular organisms. Journal of Molecular Evolution.

[ref-32] Sharp PM, Li WH (1987). The codon adaptation index-a measure of directional synonymous codon usage bias, and its potential applications. Nucleic Acids Research.

[ref-33] Shinozaki K, Shinozaki KY (1997). Gene expression and signal transduction in water- stress response. Plant Physiology.

[ref-34] Sivaram MV, Saporita JA, Furgason ML, Boettcher AJ, Munson M (2005). Dimerization of the exocyst protein Sec6p and its interaction with the t-SNARE Sec9p. Biochemistry.

[ref-35] Synek L, Schlager N, Elias M, Quentin M, Hauser MT, Zarsky V (2006). AtEXO70A1, a member of a family of putative exocyst subunits specifically expanded in land plants, is important for polar growth and plant development. The Plant Journal.

[ref-36] Teixeira FK, Menezes-benavente L, Margis R, Margis-pinheiro M (2004). Analysis of the molecular evolutionary history of the ascorbate peroxidase gene family: inferences from the rice genome. Journal of Molecular Evolution.

[ref-37] TerBush DR, Maurice T, Roth D, Novick P (1996). The exocyst is a multiprotein complex required for exocytosis in Saccharomyces cerevisiae. European Molecular Biology Organization.

[ref-38] TerBush DR, Novick P (1995). Sec6, Sec8, and Sec15 are components of a multisubunit complex which localizes to small bud tips in Saccharomyces cerevisiae. Jorunal of Cell Biology.

[ref-39] Udvardi MK, Czechowski T, Scheible WR (2008). Eleven golden rules of quantitative RT-PCR. The Plant Cell.

[ref-40] Wang Z, Li P, Yang Y, Chi Y, Fan B, Chen Z (2016). Expression and functional analysis of a novel group of legume-specific wrky and exo70 protein variants from soybean. Entific Reports.

[ref-41] Whyte JRC, Munro S (2002). Vesicle tethering complexes in membrane traffic. Journal of Cell Science.

[ref-42] Wright F (1990). The effective number of codons used in a gene. Gene.

[ref-43] Wu XM, Wu SF, Ren DM, Zhu YP, He FC (2007). The analysis method and progress in the study of codon bias. Hereditas.

[ref-44] Wu H, Turner C, Gardner J, Temple B, Brennwald P (2010). The Exo70 subunit of the exocyst is an effector for both Cdc42 and Rho3 function in polarized exocytosis. Molecular Cell Biology.

[ref-45] Yadav CB, Bonthala VS, Muthamilarasan M, Pandey G, Khan Y, Prasad M (2015). Genome-wide development of transposable elements-based markers in foxtail millet and construction of, an integrated database. DNA Research.

[ref-46] Yang Z, Bielawski JP (2000). Statistical methods for detecting molecular adaptation. Trends in Ecology & Evolution.

[ref-47] Yang K, Lu J, Zhang Y, Zhao YB, Zhang HC, Han X, Xu JH, Lu HX, Shi SM (2015b). EXO70 gene duplication and divergent evolution in Brassica oleracea and B.rapa. Science Bulletin.

[ref-48] Yang JJ, Yu DL, Chu W, Li H, Li SP (2013). Research advance of the EXO70 gene family in plants. Chinese Journal of Plant Physiology.

[ref-49] Yang K, Zhang Y, Lu J, Zhao YB, Zhang HC, Han X, He GH (2015a). Comparison of EXO70 duplication between *Arabidopsis thaliana* and *Oryza sativa*. Science Bulletin.

[ref-50] Yao XM, Wu QH, An LG, Yin M (2013). Advance in subunit of exocyst complex—Exo70. Chemistry of Life.

[ref-51] Yu D, Overdijk EJR, Berg JA, Francine G, Klaas B (2018). Solanaceous exocyst subunits are involved in immunity to diverse plant pathogens. Journal of Experimental Botany.

[ref-52] Yun HS, Kwon C (2017). Vesicle trafficking in plant immunity. Current Opinion in Plant Biology.

[ref-53] Zhang L, Guo Y, Luo L, Wang YP, Dong ZM, Sun SH, Qiu LJ (2011). Analysis of nuclear gene codon bias on soybean genome and transcriptome. Acta Agronomica Sinica.

[ref-54] Zhang Y, Liu CM, Emons AMC, Ketelaar T (2010). The plant exocyst. Journal of Integrative Plant Biology.

[ref-55] Zhang X, Orlando K, He B, Xi FG, Zhang J, Zajac A, Guo W (2008). Membrane association and functional regulation of Sec3 by phospholipids and Cdc42. Jorunal of Cell Biology.

[ref-56] Zhao J, Zhang X, Wan WT, Zhang H, Liu J, Li ML, Wang HY, Xiao J, Wang X (2018). Identification and characterization of the EXO70 gene family in polyploid wheat and related species. International Journal of Molecular Sciences.

[ref-57] Zhu YY, Wu B, Guo W (2019). The role of Exo70 in exocytosis and beyond. Small GTPases.

