# Supplementary figures and images for: Genome-wide identification and expression analysis of the EXO70 gene family in grape (Vitis vinifera L)
Source: PeerJ. 2021 Apr 21;9:e11176. doi: 10.7717/peerj.11176 (PMC8067907; doi:10.7717/peerj.11176)

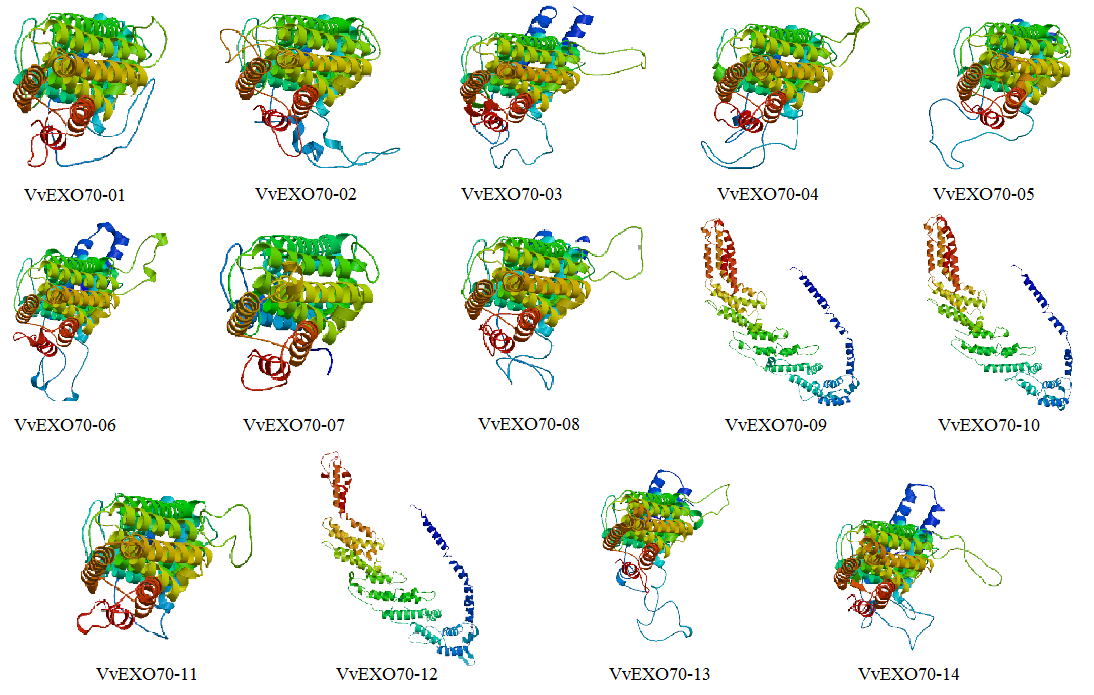

Supplement: Supplemental Information 1 [file peerj-09-11176-s001.tif]
