# Supplementary material for: Genome-wide identification and expression analysis of the EXO70 gene family in grape (Vitis vinifera L)
Source: PeerJ. 2021 Apr 21;9:e11176. doi: 10.7717/peerj.11176 (PMC8067907; doi:10.7717/peerj.11176)
Supplement: Supplemental Information 2 [file peerj-09-11176-s002.doc]

Supplementary Table S1 Numbers of genes encoding EXO70 in plant genomes

| Plant | SEC3 | SEC5 | SEC6 | SEC8 | SEC10 | SEC15 | EXO70 | EXO84 |
| --- | --- | --- | --- | --- | --- | --- | --- | --- |
| *Arabidopsis thaliana* | 2 | 2 | 1 | 1 | 1 | 2 | 23 | 3 |
| *Populus trichocarpa* | 2 | 2 | 2 | 2 | 2 | 5 | 29 | 8 |
| *Oryza sativa* | 2 | 1 | 1 | 1 | 1 | 4 | 47 | 3 |
| *Sorghum bicolor* | 2 | 1 | 1 | 1 | 1 | 3 | 31 | 3 |
| *Selaginella tamariscina* | 2 | 1 | 2 | 2 | 2 | 1 | 8 | 2 |
| *Physcomitrella patens* | 3 | 3 | 1 | 3 | 3 | 2 | 13 | 7 |
| *Nicotiana tabacum* | 4 | 4 | 2 | 2 | 2 | 4 | 44 | 6 |
| *Solanum tuberosum* | 2 | 2 | 1 | 1 | 1 | 2 | 21 | 3 |
| *Lycopersicon esculentum* | 2 | 2 | 1 | 1 | 1 | 2 | 22 | 3 |
