# Supplementary material for: Genome-wide identification and expression analysis of the EXO70 gene family in grape (Vitis vinifera L)
Source: PeerJ. 2021 Apr 21;9:e11176. doi: 10.7717/peerj.11176 (PMC8067907; doi:10.7717/peerj.11176)
Supplement: Supplemental Information 3 [file peerj-09-11176-s003.doc]

Supplementary Table S2 qRT-PCR primers for expression on analysis of VvEXO70 gene family

| Gene | Forward primer for qRT-PCR(5'-3') | Reverse primer for qRT-PCR(5'-3') |
| --- | --- | --- |
| *VvEXO70-01* | 5'- GCGTTCAAGGAGGCAACTAACAAG-3' | 5'- GGGAAGAGGATGAGGGAGCAGTG-3' |
| *VvEXO70-02* | 5'- AGAGAGCGGCGATGATGAGAGAG-3' | 5'- CATGGCAGTTTCGAGGGCAGAG-3' |
| *VvEXO70-03* | 5'- GAGGATGTGCTGAGGATGGATTGG-3' | 5'- AGATGTGGTCGCATAGCCGTTTC-3' |
| *VvEXO70-04* | 5'- ACGAGGCTGGAGGACGAGTTC-3' | 5'- CGAGAGTGAAACCCGCCGAATC-3' |
| *VvEXO70-05* | 5'- CCTTCGACCACCGCCTCTCC-3' | 5'- ATCAATTCAGCCGCCTTCAGTGTC-3' |
| *VvEXO70-06* | 5'- ACTCGTCCAAATCGCCAGTTGC-3' | 5'- GCCAATGATGTCGCCAAGAATGC-3' |
| *VvEXO70-07* | 5'- CTTGCCGCTCCCGACAAACTC-3' | 5'- CACCTCCAGATTGCTCTCCAACAG-3' |
| *VvEXO70-08* | 5'- CGCCTTCATCCTCGCACCAATC-3' | 5'- TCGTCGGAGTACCACTTGGAGATG-3' |
| *VvEXO70-09* | 5'- CGAATCCCAACCAGGCATCTCATC-3' | 5'- GTAGTCAACGACGAAGCTCACCAG-3' |
| *VvEXO70-10* | 5'- AGAGGGTGATTGATGGTGCTTGTG-3' | 5'- GAACACTGCCATTGGAAGGAGGAG-3' |
| *VvEXO70-11* | 5'- ACGCTCATGCCATCATCACCAAG-3' | 5'- AAACTCCCTGCCCTCCTTCCTG-3' |
| *VvEXO70-12* | 5'- GCAGGCAGTTGTTCCAGTCTACC-3' | 5'- TTGCTGGCTTAGGCTGAAAGAGAG-3' |
| *VvEXO70-13* | 5'- TGGGTGAGGGTGCTGTATTGTTTG-3' | 5'- CCACATTGCCTGGGTCCTATGAAC-3' |
| *VvEXO70-14* | 5'- GGCAAGGCTTGAGGAAGAGTTCAG-3' | 5'- TGAGCCCTCGTCCACCACATC-3' |
