# Supplementary material for: Genome-wide identification and expression analysis of the EXO70 gene family in grape (Vitis vinifera L)
Source: PeerJ. 2021 Apr 21;9:e11176. doi: 10.7717/peerj.11176 (PMC8067907; doi:10.7717/peerj.11176)
Supplement: Supplemental Information 4 [file peerj-09-11176-s004.doc]

**Supplementary Table S3 The secondary structure of VvEXO70 protein sequences**

| Protein | Alpha helix（%） | Beta turn（%） | Random coil（%） |
| --- | --- | --- | --- |
| VvEXO70-01 | 48.84 | 1.74 | 40.99 |
| VvEXO70-02 | 37.29 | 6.18 | 45.61 |
| VvEXO70-03 | 36.33 | 7.76 | 46.53 |
| VvEXO70-04 | 41.06 | 6.91 | 41.87 |
| VvEXO70-05 | 39.79 | 7.61 | 39.10 |
| VvEXO70-06 | 38.44 | 5.21 | 42.02 |
| VvEXO70-07 | 34.62 | 7.69 | 41.35 |
| VvEXO70-08 | 61.03 | 1.61 | 33.49 |
| VvEXO70-09 | 75.70 | 3.22 | 18.45 |
| VvEXO70-10 | 77.44 | 2.74 | 17.23 |
| VvEXO70-11 | 63.50 | 2.08 | 28.19 |
| VvEXO70-12 | 76.27 | 2.85 | 18.35 |
| VvEXO70-13 | 62.85 | 2.13 | 31.91 |
| VvEXO70-14 | 66.10 | 1.84 | 29.29 |
