# Supplementary material for: Genome-wide identification and expression analysis of the EXO70 gene family in grape (Vitis vinifera L)
Source: PeerJ. 2021 Apr 21;9:e11176. doi: 10.7717/peerj.11176 (PMC8067907; doi:10.7717/peerj.11176)
Supplement: Supplemental Information 5 [file peerj-09-11176-s005.doc]

| Gene | Cytoplasm | Chloroplast | Nucleus | Mitochonria | Peroxysome | Cytoskeleton | Plasma membrane | Extracellular matrix | Nuclear and Cytoplasmic | Nuclear and Plasma membrane |
| --- | --- | --- | --- | --- | --- | --- | --- | --- | --- | --- |
| *VvEXO70-01* | 1 | 1 | 9 |  | 2 |  |  |  |  |  |
| *VvEXO70-02* | 7 |  | 6 |  |  |  |  |  |  |  |
| *VvEXO70-03* | 4 |  | 5 | 1 | 1 | 1 | 1 |  |  |  |
| *VvEXO70-04* | 5.5 | 1 | 4.5 |  |  | 2 |  |  | 5.5 |  |
| *VvEXO70-05* | 8 |  | 4 |  |  |  |  |  |  | 3.5 |
| *VvEXO70-06* | 1 | 4 | 3 | 1 |  |  | 1 | 3 |  |  |
| *VvEXO70-07* | 4 |  | 9 |  |  |  |  |  |  |  |
| *VvEXO70-08* |  | 8 |  | 5 |  |  |  |  |  |  |
| *VvEXO70-09* | 5 | 2 | 4 | 1 |  |  | 1 |  |  |  |
| *VvEXO70-10* | 6 |  | 5 | 1 |  |  | 1 |  |  |  |
| *VvEXO70-11* |  |  |  |  |  |  | 10 |  |  |  |
| *VvEXO70-12* | 7 | 5 | 1 |  |  |  |  |  |  |  |
| *VvEXO70-13* | 2 | 1 | 2 | 1 |  |  | 3 | 2 |  |  |
| *VvEXO70-14* | 3 | 2 | 7 | 1 |  |  |  |  |  |  |

Supplementary Table S4 Subcellular location prediction of VvEXO70 gene family
