# Supplementary material for: Genome-wide identification and expression analysis of the EXO70 gene family in grape (Vitis vinifera L)
Source: PeerJ. 2021 Apr 21;9:e11176. doi: 10.7717/peerj.11176 (PMC8067907; doi:10.7717/peerj.11176)
Supplement: Supplemental Information 6 [file peerj-09-11176-s006.doc]

Supplementary Table S5 *cis*-acting elements existed in the 2 kb upstream region of VvEXO70 gene family

| Gene | ABRE | DRE/CRT | ERE | MYB | MYC | ARE | MBS | CGTCA-motif | TGACG-motif | LTR | AuxRR-core | WUN-motif | TC-rich repeats | O2-site | as-1 | STRE | TCA-element | TGA-element | Box-4 | circadian | G-box | CAT-box | GARE-motif | P-box |
| --- | --- | --- | --- | --- | --- | --- | --- | --- | --- | --- | --- | --- | --- | --- | --- | --- | --- | --- | --- | --- | --- | --- | --- | --- |
| *VvEXO70-01* | 1 | 0 | 4 | 1 | 5 | 6 | 0 | 2 | 2 | 0 | 0 | 3 | 1 | 0 | 2 | 1 | 2 | 0 | 3 | 0 | 2 | 0 | 0 | 3 |
| *VvEXO70-03* | 4 | 0 | 5 | 7 | 2 | 2 | 1 | 1 | 1 | 1 | 0 | 2 | 0 | 1 | 1 | 1 | 1 | 1 | 2 | 0 | 2 | 0 | 1 | 5 |
| *VvEXO70-04* | 7 | 1 | 2 | 3 | 3 | 0 | 1 | 2 | 2 | 0 | 0 | 2 | 1 | 0 | 2 | 0 | 0 | 0 | 6 | 0 | 5 | 0 | 0 | 0 |
| *VvEXO70-05* | 6 | 1 | 4 | 4 | 10 | 1 | 0 | 1 | 1 | 0 | 0 | 0 | 1 | 3 | 1 | 1 | 3 | 1 | 6 | 1 | 6 | 0 | 0 | 0 |
| *VvEXO70-06* | 8 | 0 | 3 | 7 | 3 | 3 | 0 | 1 | 1 | 0 | 2 | 1 | 1 | 0 | 1 | 2 | 1 | 1 | 2 | 0 | 8 | 1 | 1 | 1 |
| *VvEXO70-07* | 4 | 0 | 3 | 2 | 3 | 0 | 1 | 3 | 3 | 0 | 1 | 1 | 0 | 1 | 3 | 1 | 2 | 2 | 5 | 1 | 2 | 1 | 0 | 1 |
| *VvEXO70-08* | 1 | 0 | 4 | 3 | 10 | 1 | 0 | 2 | 2 | 0 | 0 | 0 | 1 | 1 | 2 | 2 | 1 | 1 | 5 | 0 | 1 | 0 | 0 | 1 |
| *VvEXO70-09* | 4 | 0 | 0 | 5 | 4 | 0 | 0 | 0 | 0 | 0 | 0 | 0 | 0 | 0 | 0 | 0 | 0 | 0 | 1 | 0 | 2 | 1 | 0 | 0 |
| *VvEXO70-10* | 0 | 0 | 3 | 5 | 4 | 4 | 0 | 0 | 0 | 0 | 0 | 0 | 1 | 0 | 0 | 1 | 1 | 0 | 3 | 0 | 1 | 2 | 0 | 0 |
| *VvEXO70-11* | 1 | 0 | 4 | 3 | 2 | 1 | 1 | 2 | 2 | 1 | 0 | 0 | 0 | 1 | 2 | 2 | 0 | 1 | 2 | 0 | 1 | 1 | 0 | 0 |
| *VvEXO70-12* | 4 | 0 | 1 | 3 | 3 | 2 | 0 | 1 | 1 | 0 | 1 | 0 | 1 | 1 | 1 | 2 | 0 | 0 | 4 | 0 | 3 | 0 | 0 | 0 |
| *VvEXO70-13* | 2 | 0 | 4 | 2 | 1 | 2 | 0 | 0 | 0 | 0 | 0 | 3 | 0 | 0 | 0 | 0 | 1 | 0 | 2 | 0 | 2 | 0 | 0 | 0 |
| *VvEXO70-14* | 0 | 0 | 3 | 4 | 2 | 2 | 0 | 0 | 0 | 0 | 0 | 0 | 1 | 0 | 0 | 1 | 1 | 1 | 3 | 1 | 1 | 0 | 1 | 2 |

| Gene | CGTCA-motif | TGACG-motif | LTR | AuxRR-core | WUN-motif | TC-rich repeats | O2-site | as-1 | STRE |
| --- | --- | --- | --- | --- | --- | --- | --- | --- | --- |
| *VvEXO70-01* | 2 | 2 | 0 | 0 | 3 | 1 | 0 | 2 | 1 |
| *VvEXO70-03* | 1 | 1 | 1 | 0 | 2 | 0 | 1 | 1 | 1 |
| *VvEXO70-04* | 2 | 2 | 0 | 0 | 2 | 1 | 0 | 2 | 0 |
| *VvEXO70-05* | 1 | 1 | 0 | 0 | 0 | 1 | 3 | 1 | 1 |
| *VvEXO70-06* | 1 | 1 | 0 | 2 | 1 | 1 | 0 | 1 | 2 |
| *VvEXO70-07* | 3 | 3 | 0 | 1 | 1 | 0 | 1 | 3 | 1 |
| *VvEXO70-08* | 2 | 2 | 0 | 0 | 0 | 1 | 1 | 2 | 2 |
| *VvEXO70-09* | 0 | 0 | 0 | 0 | 0 | 0 | 0 | 0 | 0 |
| *VvEXO70-10* | 0 | 0 | 0 | 0 | 0 | 1 | 0 | 0 | 1 |
| *VvEXO70-11* | 2 | 2 | 1 | 0 | 0 | 0 | 1 | 2 | 2 |
| *VvEXO70-12* | 1 | 1 | 0 | 1 | 0 | 1 | 1 | 1 | 2 |
| *VvEXO70-13* | 0 | 0 | 0 | 0 | 3 | 0 | 0 | 0 | 0 |
| *VvEXO70-14* | 0 | 0 | 0 | 0 | 0 | 1 | 0 | 0 | 1 |

| Gene | TCA-element | TGA-element | Box-4 | circadian | G-box | CAT-box | GARE-motif | P-box |
| --- | --- | --- | --- | --- | --- | --- | --- | --- |
| *VvEXO70-01* | 2 | 0 | 3 | 0 | 2 | 0 | 0 | 3 |
| *VvEXO70-03* | 1 | 1 | 2 | 0 | 2 | 0 | 1 | 5 |
| *VvEXO70-04* | 0 | 0 | 6 | 0 | 5 | 0 | 0 | 0 |
| *VvEXO70-05* | 3 | 1 | 6 | 1 | 6 | 0 | 0 | 0 |
| *VvEXO70-06* | 1 | 1 | 2 | 0 | 8 | 1 | 1 | 1 |
| *VvEXO70-07* | 2 | 2 | 5 | 1 | 2 | 1 | 0 | 1 |
| *VvEXO70-08* | 1 | 1 | 5 | 0 | 1 | 0 | 0 | 1 |
| *VvEXO70-09* | 0 | 0 | 1 | 0 | 2 | 1 | 0 | 0 |
| *VvEXO70-10* | 1 | 0 | 3 | 0 | 1 | 2 | 0 | 0 |
| *VvEXO70-11* | 0 | 1 | 2 | 0 | 1 | 1 | 0 | 0 |
| *VvEXO70-12* | 0 | 0 | 4 | 0 | 3 | 0 | 0 | 0 |
| *VvEXO70-13* | 1 | 0 | 2 | 0 | 2 | 0 | 0 | 0 |
| *VvEXO70-14* | 1 | 1 | 3 | 1 | 1 | 0 | 1 | 2 |
