# Supplementary material for: Genome-wide identification and expression analysis of the EXO70 gene family in grape (Vitis vinifera L)
Source: PeerJ. 2021 Apr 21;9:e11176. doi: 10.7717/peerj.11176 (PMC8067907; doi:10.7717/peerj.11176)
Supplement: Supplemental Information 7 [file peerj-09-11176-s007.doc]

Supplementary Table S6 The Codon number and RSCU value of VvEXO70 gene family

| amino acid | Codon | VvEX70-01 | | VvEX70-02 | | VvEX70-03 | | VvEX70-04 | | VvEX70-05 | | VvEX70-06 | | VvEX70-07 | |
| --- | --- | --- | --- | --- | --- | --- | --- | --- | --- | --- | --- | --- | --- | --- | --- |
|  |  | number | RSCU | number | RSCU | number | RSCU | number | RSCU | number | RSCU | number | RSCU | number | RSCU |
| Phe | UUU | 7 | 0.47 | 22 | 1.42 | 15 | 1.25 | 10 | 0.91 | 19 | 1.41 | 11 | 0.69 | 5 | 0.50 |
|  | UUC | 23 | 1.53 | 9 | 0.58 | 9 | 0.75 | 12 | 1.09 | 8 | 0.59 | 21 | 1.31 | 15 | 1.50 |
| Leu | UUA | 0 | 0.00 | 11 | 0.96 | 4 | 0.32 | 4 | 0.35 | 11 | 0.92 | 9 | 0.35 | 12 | 1.41 |
|  | UUG | 8 | 0.94 | 14 | 1.22 | 22 | 1.74 | 31 | 2.70 | 15 | 1.25 | 10 | 1.05 | 12 | 1.41 |
|  | CUU | 5 | 0.59 | 11 | 0.96 | 16 | 1.26 | 9 | 0.78 | 11 | 0.92 | 13 | 1.37 | 7 | 0.82 |
|  | CUC | 12 | 1.41 | 11 | 0.96 | 15 | 1.18 | 12 | 1.04 | 13 | 1.08 | 16 | 1.68 | 8 | 0.94 |
|  | CUA | 6 | 0.71 | 6 | 0.52 | 7 | 0.55 | 3 | 0.26 | 8 | 0.67 | 2 | 0.21 | 1 | 0.12 |
|  | CUG | 20 | 2.35 | 16 | 1.39 | 12 | 0.95 | 10 | 0.87 | 14 | 1.17 | 7 | 0.74 | 11 | 1.29 |
| Ser | UCU | 11 | 0.89 | 18 | 1.80 | 13 | 1.37 | 5 | 0.58 | 14 | 1.62 | 25 | 1.53 | 6 | 0.95 |
|  | UCC | 21 | 1.70 | 6 | 0.60 | 8 | 0.84 | 7 | 0.81 | 5 | 0.58 | 24 | 1.47 | 8 | 1.26 |
|  | UCA | 7 | 0.57 | 10 | 1.00 | 15 | 1.58 | 10 | 1.15 | 11 | 1.27 | 14 | 0.86 | 7 | 1.11 |
|  | UCG | 12 | 0.97 | 1 | 0.10 | 4 | 0.42 | 11 | 1.27 | 2 | 0.23 | 20 | 1.22 | 5 | 0.79 |
|  | AGU | 5 | 0.41 | 12 | 1.20 | 7 | 0.74 | 13 | 1.50 | 11 | 1.27 | 6 | 0.37 | 5 | 0.79 |
|  | AGC | 18 | 1.46 | 13 | 1.30 | 10 | 1.05 | 6 | 0.69 | 9 | 1.04 | 9 | 0.55 | 7 | 1.11 |
| Pro | CCU | 7 | 0.93 | 7 | 1.47 | 7 | 1.75 | 1 | 0.33 | 10 | 1.90 | 10 | 1.11 | 3 | 0.86 |
|  | CCC | 4 | 0.53 | 4 | 0.84 | 1 | 0.25 | 4 | 1.33 | 3 | 0.57 | 6 | 0.67 | 5 | 1.43 |
|  | CCA | 8 | 1.07 | 8 | 1.68 | 7 | 1.75 | 2 | 0.67 | 7 | 1.33 | 9 | 1.00 | 3 | 0.86 |
|  | CCG | 11 | 1.47 | 0 | 0.00 | 1 | 0.25 | 5 | 1.67 | 1 | 0.19 | 11 | 1.22 | 3 | 0.86 |
| Tyr | UAU | 3 | 0.30 | 7 | 1.00 | 10 | 1.0 | 11 | 1.16 | 7 | 0.82 | 11 | 0.96 | 4 | 0.73 |
|  | UAC | 17 | 1.70 | 7 | 1.00 | 10 | 1.0 | 8 | 0.84 | 10 | 1.18 | 12 | 1.04 | 7 | 1.27 |
| Cys | UGU | 3 | 0.67 | 3 | 1.50 | 3 | 0.75 | 8 | 1.45 | 5 | 1.67 | 1 | 0.25 | 3 | 0.60 |
|  | UGC | 6 | 1.33 | 1 | 0.50 | 5 | 1.25 | 3 | 0.55 | 1 | 0.33 | 7 | 1.75 | 7 | 1.40 |
| Trp | UGG | 5 | 1.00 | 5 | 1.00 | 7 | 1.00 | 0 | 1.00 | 5 | 1.00 | 7 | 1.00 | 7 | 1.00 |
| His | CAU | 6 | 0.75 | 11 | 1.29 | 8 | 0.80 | 3 | 0.67 | 11 | 1.05 | 7 | 0.88 | 4 | 1.00 |
|  | CAC | 10 | 1.25 | 6 | 0.71 | 12 | 1.20 | 6 | 1.33 | 10 | 0.95 | 9 | 1.13 | 4 | 1.00 |
| Gln | CAA | 10 | 0.87 | 17 | 0.85 | 8 | 0.70 | 8 | 0.73 | 24 | 1.17 | 8 | 0.89 | 9 | 1.00 |
|  | CAG | 13 | 1.13 | 23 | 1.15 | 15 | 1.30 | 14 | 1.27 | 17 | 0.83 | 10 | 1.11 | 9 | 1.00 |
| Arg | CGU | 4 | 0.71 | 8 | 1.14 | 3 | 0.53 | 5 | 0.67 | 3 | 0.49 | 8 | 1.17 | 2 | 0.32 |
|  | CGC | 4 | 0.71 | 1 | 0.14 | 2 | 0.35 | 7 | 0.93 | 3 | 0.49 | 6 | 0.88 | 3 | 0.47 |
|  | CGA | 8 | 1.41 | 9 | 1.29 | 2 | 0.35 | 3 | 0.40 | 5 | 0.81 | 7 | 1.02 | 7 | 1.11 |
|  | CGG | 6 | 1.06 | 4 | 0.57 | 6 | 1.06 | 8 | 1.07 | 4 | 0.65 | 7 | 1.02 | 8 | 1.26 |
|  | AGA | 4 | 0.71 | 12 | 1.71 | 8 | 1.41 | 8 | 1.07 | 9 | 1.46 | 10 | 1.46 | 11 | 1.74 |
|  | AGG | 8 | 1.41 | 8 | 1.14 | 13 | 2.29 | 14 | 1.87 | 13 | 2.11 | 3 | 0.44 | 7 | 1.11 |
| Ile | AUU | 12 | 1.20 | 21 | 2.10 | 21 | 1.34 | 26 | 2.11 | 21 | 1.91 | 15 | 1.02 | 10 | 0.97 |
|  | AUC | 16 | 1.60 | 3 | 0.30 | 17 | 1.09 | 7 | 0.57 | 3 | 0.27 | 16 | 1.09 | 19 | 1.84 |
|  | AUA | 2 | 0.20 | 6 | 0.60 | 9 | 0.57 | 4 | 0.32 | 9 | 0.82 | 13 | 0.89 | 2 | 0.19 |
| Met | AUG | 19 | 1.00 | 17 | 1.00 | 18 | 1.00 | 21 | 1.00 | 16 | 1.00 | 23 | 1.0 | 12 | 1.00 |
| Thr | ACU | 5 | 0.67 | 14 | 1.65 | 7 | 1.40 | 5 | 0.71 | 11 | 1.29 | 11 | 1.42 | 4 | 1.14 |
|  | ACC | 11 | 1.47 | 7 | 0.82 | 6 | 1.20 | 5 | 0.71 | 9 | 1.06 | 5 | 0.65 | 7 | 2.00 |
|  | ACA | 9 | 1.20 | 11 | 1.29 | 4 | 0.80 | 9 | 1.29 | 10 | 1.18 | 9 | 1.16 | 2 | 0.57 |
|  | ACG | 5 | 0.67 | 2 | 0.24 | 3 | 0.60 | 9 | 1.29 | 4 | 0.47 | 6 | 0.77 | 1 | 0.29 |
| Asn | AAU | 6 | 0.57 | 16 | 1.23 | 19 | 1.27 | 14 | 1.08 | 12 | 1.04 | 12 | 1.20 | 9 | 0.95 |
|  | AAC | 15 | 1.43 | 10 | 0.77 | 11 | 0.73 | 12 | 0.92 | 11 | 0.96 | 8 | 0.80 | 10 | 1.05 |
| Lys | AAA | 19 | 0.90 | 18 | 0.86 | 15 | 0.71 | 9 | 0.53 | 20 | 0.93 | 23 | 1.10 | 9 | 0.90 |
|  | AAG | 23 | 1.10 | 24 | 1.14 | 27 | 1.29 | 25 | 1.47 | 23 | 1.07 | 19 | 0.90 | 11 | 1.10 |
| Val | GUU | 7 | 0.76 | 18 | 1.85 | 11 | 1.26 | 16 | 1.60 | 12 | 1.41 | 6 | 0.73 | 10 | 1.48 |
|  | GUC | 8 | 0.86 | 4 | 0.41 | 6 | 0.69 | 5 | 0.50 | 7 | 0.82 | 9 | 1.09 | 6 | 0.89 |
|  | GUA | 2 | 0.22 | 4 | 0.41 | 6 | 0.69 | 1 | 0.10 | 5 | 0.59 | 5 | 0.61 | 1 | 0.15 |
|  | GUG | 20 | 2.16 | 13 | 1.33 | 12 | 1.37 | 18 | 1.80 | 10 | 1.18 | 13 | 1.58 | 10 | 1.48 |
| Ala | GCU | 6 | 0.63 | 18 | 1.53 | 15 | 1.58 | 8 | 0.67 | 24 | 1.81 | 6 | 0.48 | 7 | 0.97 |
|  | GCC | 7 | 0.74 | 9 | 0.77 | 4 | 0.42 | 17 | 1.42 | 12 | 0.91 | 20 | 1.60 | 6 | 0.83 |
|  | GCA | 16 | 1.68 | 14 | 1.19 | 16 | 1.68 | 9 | 0.75 | 13 | 0.98 | 11 | 0.88 | 10 | 1.38 |
|  | GCG | 9 | 0.95 | 6 | 0.51 | 3 | 0.32 | 14 | 1.17 | 4 | 0.30 | 13 | 1.04 | 6 | 0.83 |
| Asp | GAU | 22 | 0.98 | 20 | 1.25 | 23 | 1.48 | 31 | 1.32 | 25 | 1.67 | 21 | 1.11 | 11 | 0.96 |
|  | GAC | 23 | 1.02 | 12 | 0.75 | 8 | 0.52 | 16 | 0.68 | 5 | 0.33 | 17 | 0.89 | 12 | 1.04 |
| Glu | GAA | 19 | 0.67 | 28 | 1.12 | 33 | 1.02 | 18 | 0.57 | 30 | 1.20 | 23 | 0.98 | 16 | 0.89 |
|  | GAG | 38 | 1.33 | 22 | 0.88 | 32 | 0.98 | 45 | 1.43 | 20 | 0.80 | 24 | 1.02 | 20 | 1.11 |
| Gly | GGU | 6 | 0.86 | 12 | 1.50 | 12 | 1.50 | 12 | 1.12 | 15 | 1.82 | 4 | 0.67 | 7 | 1.04 |
|  | GGC | 5 | 0.71 | 5 | 0.63 | 4 | 0.50 | 7 | 0.65 | 3 | 0.36 | 9 | 1.50 | 7 | 1.04 |
|  | GGA | 10 | 1.43 | 5 | 0.63 | 8 | 1.00 | 10 | 0.93 | 5 | 0.61 | 9 | 1.50 | 9 | 1.33 |
|  | GGG | 7 | 1.00 | 10 | 1.25 | 8 | 1.00 | 14 | 1.30 | 10 | 1.21 | 2 | 0.33 | 4 | 0.59 |

| amino acid | Codon | VvEX70-08 | | VvEX70-09 | | VvEX70-10 | | VvEX70-11 | | VvEX70-12 | | VvEX70-13 | | VvEX70-14 | |
| --- | --- | --- | --- | --- | --- | --- | --- | --- | --- | --- | --- | --- | --- | --- | --- |
|  |  | number | RSCU | number | RSCU | number | RSCU | number | RSCU | number | RSCU | number | RSCU | number | RSCU |
| Phe | UUU | 11 | 0.73 | 16 | 1.19 | 15 | 1.20 | 21 | 1.08 | 15 | 1.25 | 14 | 1.12 | 17 | 1.48 |
|  | UUC | 19 | 1.27 | 11 | 0.81 | 10 | 0.80 | 18 | 0.92 | 9 | 0.75 | 11 | 0.88 | 6 | 0.52 |
| Leu | UUA | 1 | 0.11 | 5 | 0.35 | 6 | 0.40 | 7 | 0.55 | 9 | 0.56 | 6 | 0.62 | 6 | 0.50 |
|  | UUG | 9 | 1.00 | 25 | 1.74 | 24 | 1.60 | 22 | 1.71 | 35 | 2.16 | 23 | 2.38 | 23 | 1.92 |
|  | CUU | 8 | 0.89 | 14 | 0.98 | 14 | 0.93 | 3 | 0.23 | 24 | 1.48 | 8 | 0.83 | 17 | 1.42 |
|  | CUC | 26 | 2.89 | 16 | 1.12 | 17 | 1.13 | 25 | 1.95 | 10 | 0.62 | 7 | 0.72 | 12 | 1.00 |
|  | CUA | 5 | 0.56 | 3 | 0.21 | 7 | 0.47 | 7 | 0.55 | 8 | 0.49 | 2 | 0.21 | 6 | 0.50 |
|  | CUG | 5 | 0.56 | 23 | 1.60 | 22 | 1.47 | 13 | 1.01 | 11 | 0.68 | 12 | 1.24 | 8 | 0.67 |
| Ser | UCU | 19 | 1.15 | 12 | 1.20 | 9 | 0.96 | 19 | 1.30 | 8 | 0.98 | 9 | 0.86 | 15 | 1.38 |
|  | UCC | 28 | 1.70 | 9 | 0.90 | 11 | 1.18 | 17 | 1.16 | 8 | 0.98 | 4 | 0.38 | 9 | 0.83 |
|  | UCA | 15 | 0.91 | 11 | 1.10 | 9 | 0.96 | 31 | 2.11 | 7 | 0.86 | 14 | 1.33 | 15 | 1.38 |
|  | UCG | 26 | 1.58 | 6 | 0.60 | 3 | 0.32 | 10 | 0.68 | 5 | 0.61 | 12 | 1.14 | 3 | 0.28 |
|  | AGU | 3 | 0.18 | 12 | 1.20 | 15 | 1.61 | 9 | 0.61 | 13 | 1.59 | 13 | 1.24 | 17 | 1.57 |
|  | AGC | 8 | 0.48 | 10 | 1.00 | 9 | 0.96 | 2 | 0.14 | 8 | 0.98 | 11 | 1.05 | 6 | 0.55 |
| Pro | CCU | 5 | 0.87 | 7 | 1.40 | 5 | 1.25 | 5 | 0.91 | 9 | 1.44 | 1 | 0.20 | 7 | 1.33 |
|  | CCC | 3 | 0.52 | 3 | 0.60 | 3 | 0.75 | 3 | 0.55 | 5 | 0.80 | 3 | 0.60 | 2 | 0.38 |
|  | CCA | 9 | 1.57 | 7 | 1.40 | 5 | 1.25 | 8 | 1.45 | 9 | 1.44 | 7 | 1.40 | 11 | 2.10 |
|  | CCG | 6 | 1.04 | 3 | 0.60 | 3 | 0.75 | 6 | 1.09 | 2 | 0.32 | 9 | 1.80 | 1 | 0.19 |
| Tyr | UAU | 5 | 0.42 | 10 | 0.80 | 10 | 0.80 | 15 | 1.11 | 9 | 1.00 | 7 | 0.74 | 9 | 1.00 |
|  | UAC | 19 | 1.58 | 15 | 1.20 | 15 | 1.20 | 12 | 0.89 | 9 | 1.00 | 12 | 1.26 | 9 | 1.00 |
| Cys | UGU | 4 | 1.14 | 6 | 0.92 | 7 | 0.93 | 8 | 1.14 | 5 | 1.11 | 3 | 0.75 | 6 | 1.20 |
|  | UGC | 3 | 0.86 | 7 | 1.08 | 8 | 1.07 | 6 | 0.86 | 4 | 0.89 | 5 | 1.25 | 4 | 0.80 |
| Trp | UGG | 9 | 1.00 | 7 | 1.00 | 7 | 1.00 | 8 | 1.00 | 9 | 1.00 | 7 | 1.00 | 9 | 1.00 |
| His | CAU | 8 | 1.45 | 8 | 1.07 | 8 | 1.07 | 9 | 1.13 | 6 | 1.09 | 6 | 0.92 | 6 | 0.75 |
|  | CAC | 3 | 0.55 | 7 | 0.93 | 7 | 0.93 | 7 | 0.88 | 5 | 0.91 | 7 | 1.08 | 10 | 1.25 |
| Gln | CAA | 7 | 0.88 | 5 | 0.43 | 7 | 0.61 | 10 | 1.00 | 11 | 0.88 | 7 | 0.67 | 5 | 0.45 |
|  | CAG | 9 | 1.13 | 18 | 1.57 | 16 | 1.39 | 10 | 1.00 | 14 | 1.12 | 14 | 1.33 | 17 | 1.55 |
| Arg | CGU | 5 | 0.83 | 4 | 0.63 | 2 | 0.35 | 3 | 0.60 | 2 | 0.38 | 2 | 0.24 | 2 | 0.35 |
|  | CGC | 6 | 1.00 | 3 | 0.43 | 2 | 0.35 | 1 | 0.20 | 2 | 0.38 | 3 | 0.36 | 4 | 0.71 |
|  | CGA | 4 | 0.67 | 1 | 0.16 | 0 | 0.00 | 1 | 0.20 | 5 | 0.94 | 5 | 0.60 | 1 | 0.18 |
|  | CGG | 3 | 0.50 | 6 | 0.95 | 4 | 0.71 | 6 | 1.20 | 4 | 0.75 | 15 | 1.80 | 8 | 1.41 |
|  | AGA | 9 | 1.50 | 6 | 0.95 | 8 | 1.41 | 7 | 1.40 | 9 | 1.69 | 12 | 1.44 | 9 | 1.59 |
|  | AGG | 9 | 1.50 | 18 | 2.84 | 18 | 3.18 | 12 | 2.40 | 10 | 1.88 | 13 | 1.56 | 10 | 1.76 |
| Ile | AUU | 14 | 1.27 | 23 | 1.68 | 21 | 1.62 | 19 | 1.21 | 16 | 1.37 | 20 | 1.20 | 19 | 1.46 |
|  | AUC | 17 | 1.55 | 12 | 0.88 | 11 | 0.85 | 18 | 1.15 | 11 | 0.94 | 17 | 1.02 | 8 | 0.62 |
|  | AUA | 2 | 0.18 | 6 | 0.44 | 7 | 0.54 | 10 | 0.64 | 8 | 0.69 | 13 | 0.78 | 12 | 0.98 |
| Met | AUG | 16 | 1.00 | 12 | 1.00 | 12 | 1.00 | 17 | 1.00 | 9 | 1.00 | 11 | 1.00 | 15 | 1.00 |
| Thr | ACU | 3 | 0.60 | 10 | 1.60 | 12 | 2.09 | 6 | 1.14 | 5 | 1.33 | 10 | 1.54 | 6 | 1.00 |
|  | ACC | 7 | 1.40 | 9 | 1.44 | 7 | 1.22 | 8 | 1.52 | 2 | 0.53 | 6 | 0.92 | 8 | 1.33 |
|  | ACA | 6 | 1.20 | 5 | 0.80 | 3 | 0.52 | 6 | 1.14 | 7 | 1.87 | 4 | 0.62 | 7 | 1.17 |
|  | ACG | 4 | 0.80 | 1 | 0.16 | 1 | 0.17 | 1 | 0.19 | 1 | 0.27 | 6 | 0.92 | 3 | 0.50 |
| Asn | AAU | 8 | 0.89 | 15 | 0.97 | 19 | 1.23 | 11 | 1.16 | 18 | 1.29 | 8 | 1.23 | 17 | 1.03 |
|  | AAC | 10 | 1.11 | 16 | 1.03 | 12 | 0.77 | 8 | 0.84 | 10 | 0.71 | 5 | 0.77 | 16 | 0.97 |
| Lys | AAA | 17 | 0.83 | 18 | 0.75 | 18 | 0.77 | 16 | 0.74 | 18 | 0.80 | 8 | 0.62 | 13 | 0.76 |
|  | AAG | 24 | 1.17 | 30 | 1.25 | 29 | 1.23 | 27 | 1.26 | 27 | 1.20 | 18 | 1.38 | 21 | 1.24 |
| Val | GUU | 8 | 0.86 | 20 | 1.74 | 11 | 1.10 | 12 | 1.26 | 19 | 2.00 | 8 | 0.97 | 18 | 1.71 |
|  | GUC | 14 | 1.51 | 7 | 0.61 | 10 | 1.00 | 9 | 0.95 | 6 | 0.63 | 5 | 0.61 | 7 | 0.67 |
|  | GUA | 3 | 0.32 | 5 | 0.43 | 6 | 0.60 | 10 | 1.05 | 2 | 0.21 | 3 | 0.36 | 4 | 0.38 |
|  | GUG | 12 | 1.30 | 14 | 1.22 | 13 | 1.30 | 7 | 0.74 | 11 | 1.16 | 17 | 2.06 | 13 | 1.24 |
| Ala | GCU | 10 | 0.95 | 16 | 1.39 | 16 | 1.49 | 13 | 1.24 | 16 | 1.31 | 18 | 1.57 | 16 | 1.68 |
|  | GCC | 14 | 1.33 | 13 | 1.13 | 12 | 1.12 | 17 | 1.62 | 7 | 0.57 | 9 | 0.78 | 5 | 0.53 |
|  | GCA | 5 | 0.48 | 14 | 1.22 | 15 | 1.40 | 5 | 0.48 | 16 | 1.31 | 8 | 0.70 | 16 | 1.68 |
|  | GCG | 13 | 1.24 | 3 | 0.26 | 0 | 0.00 | 7 | 0.67 | 10 | 0.82 | 11 | 0.96 | 1 | 0.11 |
| Asp | GAU | 18 | 1.09 | 22 | 1.29 | 21 | 1.24 | 13 | 0.87 | 28 | 1.75 | 27 | 1.42 | 32 | 1.36 |
|  | GAC | 15 | 0.91 | 12 | 0.71 | 13 | 0.76 | 17 | 1.13 | 4 | 0.25 | 11 | 0.58 | 15 | 0.64 |
| Glu | GAA | 22 | 0.90 | 16 | 0.64 | 7 | 0.68 | 18 | 0.78 | 18 | 0.78 | 17 | 0.60 | 24 | 0.79 |
|  | GAG | 27 | 1.10 | 34 | 1.36 | 16 | 1.32 | 28 | 1.22 | 28 | 1.22 | 40 | 1.40 | 37 | 1.21 |
| Gly | GGU | 8 | 1.39 | 6 | 0.67 | 5 | 0.65 | 3 | 0.40 | 7 | 0.78 | 8 | 1.19 | 9 | 1.24 |
|  | GGC | 3 | 0.52 | 9 | 1.00 | 9 | 1.16 | 5 | 0.67 | 4 | 0.44 | 3 | 0.44 | 3 | 0.41 |
|  | GGA | 8 | 1.39 | 9 | 1.00 | 8 | 1.03 | 10 | 1.33 | 7 | 0.78 | 6 | 0.89 | 6 | 0.83 |
|  | GGG | 4 | 0.70 | 12 | 1.33 | 9 | 1.16 | 12 | 1.60 | 18 | 2.00 | 10 | 1.48 | 11 | 1.52 |
