# Supplementary material for: Genome-wide identification and expression analysis of the EXO70 gene family in grape (Vitis vinifera L)
Source: PeerJ. 2021 Apr 21;9:e11176. doi: 10.7717/peerj.11176 (PMC8067907; doi:10.7717/peerj.11176)
Supplement: Supplemental Information 8 [file peerj-09-11176-s008.doc]

Supplementary Table S7 Codon using feature of EXO70 gene in grape

| gene | T3s | C3s | A3s | G3s | CAI | CBI | Fop | Nc | GC3s | GC | L_sym | L_aa | Gravy | Aromo |
| --- | --- | --- | --- | --- | --- | --- | --- | --- | --- | --- | --- | --- | --- | --- |
| *VvEXO70-01* | 0.2333 | 0.4057 | 0.2532 | 0.4054 | 0.267 | 0.102 | 0.486 | 53.99 | 0.618 | 0.517 | 615 | 639 | -0.55352 | 0.086072 |
| *VvEXO70-02* | 0.4395 | 0.2177 | 0.3155 | 0.3017 | 0.217 | -0.015 | 0.412 | 54.57 | 0.4 | 0.44 | 628 | 650 | -0.44338 | 0.076923 |
| *VvEXO70-03* | 0.3893 | 0.2623 | 0.2928 | 0.3607 | 0.205 | -0.043 | 0.392 | 54.98 | 0.463 | 0.439 | 618 | 643 | -0.30404 | 0.079316 |
| *VvEXO70-04* | 0.3484 | 0.2638 | 0.1988 | 0.4781 | 0.179 | -0.129 | 0.346 | 53.8 | 0.561 | 0.493 | 627 | 657 | -0.35799 | 0.076104 |
| *VvEXO70-05* | 0.428 | 0.2272 | 0.332 | 0.2915 | 0.201 | -0.061 | 0.386 | 54.19 | 0.397 | 0.44 | 627 | 648 | -0.40571 | 0.075617 |
| *VvEXO70-06* | 0.3031 | 0.3521 | 0.2917 | 0.304 | 0.213 | 0.03 | 0.441 | 58.05 | 0.515 | 0.481 | 658 | 688 | -0.34448 | 0.090116 |
| *VvEXO70-07* | 0.2694 | 0.3639 | 0.2886 | 0.3429 | 0.2 | 0.069 | 0.452 | 59.07 | 0.548 | 0.488 | 434 | 453 | -0.22495 | 0.083885 |
| *VvEXO70-08* | 0.2796 | 0.398 | 0.2389 | 0.3432 | 0.218 | 0.028 | 0.441 | 54.88 | 0.581 | 0.497 | 596 | 621 | -0.36232 | 0.101449 |
| *VvEXO70-09* | 0.3702 | 0.2928 | 0.2139 | 0.4038 | 0.213 | -0.012 | 0.405 | 53.86 | 0.53 | 0.467 | 664 | 683 | -0.17818 | 0.086384 |
| *VvEXO70-10* | 0.3675 | 0.3017 | 0.2358 | 0.3863 | 0.202 | -0.046 | 0.385 | 53.2 | 0.52 | 0.455 | 637 | 656 | -0.17043 | 0.08689 |
| *VvEXO70-11* | 0.313 | 0.3204 | 0.2897 | 0.3523 | 0.19 | -0.069 | 0.378 | 55.21 | 0.515 | 0.454 | 649 | 674 | -0.02255 | 0.109792 |
| *VvEXO70-12* | 0.4016 | 0.2088 | 0.2724 | 0.3851 | 0.164 | -0.114 | 0.336 | 54.18 | 0.456 | 0.448 | 614 | 632 | -0.10775 | 0.080696 |
| *VvEXO70-13* | 0.3313 | 0.2434 | 0.2348 | 0.4684 | 0.172 | -0.076 | 0.373 | 54.61 | 0.538 | 0.487 | 593 | 611 | -0.30147 | 0.08347 |
| *VvEXO70-14* | 0.4168 | 0.2427 | 0.2807 | 0.3529 | 0.196 | -0.093 | 0.363 | 53.21 | 0.446 | 0.444 | 628 | 652 | -0.36963 | 0.076687 |
| Average | 0.349357 | 0.292893 | 0.267057 | 0.369736 | 0.202643 | -0.03064 | 0.399714 | 54.84286 | 0.506286 | 0.467857 | 613.4286 | 636.2143 | -0.29617 | 0.085243 |
