# Supplementary material for: Genome-wide identification and expression analysis of the EXO70 gene family in grape (Vitis vinifera L)
Source: PeerJ. 2021 Apr 21;9:e11176. doi: 10.7717/peerj.11176 (PMC8067907; doi:10.7717/peerj.11176)
Supplement: Supplemental Information 9 [file peerj-09-11176-s009.doc]

Supplementary Table S8 Codon use correlation analysis of VvEXO70 gene family

|  | T3s | C3s | A3s | G3s | CAI | CBI | Fop | ENC | GC3s | GC | L_sym | L_aa | Gravy |
| --- | --- | --- | --- | --- | --- | --- | --- | --- | --- | --- | --- | --- | --- |
| C3s | -0.909^**^ |  |  |  |  |  |  |  |  |  |  |  |  |
| A3s | 0.330 | -0.223 |  |  |  |  |  |  |  |  |  |  |  |
| G3s | -0.221 | -0.059 | -0.843^**^ |  |  |  |  |  |  |  |  |  |  |
| CAI | -0.428 | 0.641^*^ | 0.081 | -0.279 |  |  |  |  |  |  |  |  |  |
| CBI | -0.672^**^ | 0.801^**^ | 0.120 | -0.326 | 0.821^**^ |  |  |  |  |  |  |  |  |
| Fop | -0.678^**^ | 0.811^**^ | 0.124 | -0.329 | 0.856^**^ | 0.990^**^ |  |  |  |  |  |  |  |
| ENC | -0.473 | 0.433 | 0.343 | -0.379 | 0.036 | 0.519 | 0.490 |  |  |  |  |  |  |
| GC3s | -0.899^**^ | 0.795^**^ | -0.703^**^ | 0.553^*^ | 0.334 | 0.465 | 0.471 | 0.159 |  |  |  |  |  |
| GC | -0.877^**^ | 0.740^**^ | -0.563^*^ | 0.457 | 0.386 | 0.541^*^ | 0.567^*^ | 0.259 | 0.913^**^ |  |  |  |  |
| L_sym | 0.380 | -0.269 | -0.117 | 0.025 | 0.068 | -0.373 | -0.317 | -0.603^*^ | -0.220 | -0.304 |  |  |  |
| L_aa | 0.354 | -0.241 | -0.116 | 0.019 | 0.081 | -0.361 | -0.300 | -0.577^*^ | -0.201 | -0.279 | 0.998^**^ |  |  |
| Gravy | 0.074 | -0.137 | -0.116 | 0.103 | -0.606^*^ | -0.380 | -0.463 | 0.100 | -0.040 | -0.329 | -0.009 | -0.032 |  |
| Aromo | -0.575^*^ | 0.620^*^ | -0.115 | -0.105 | 0.138 | 0.274 | 0.280 | 0.227 | 0.466 | 0.269 | 0.092 | 0.101 | 0.445 |
